# A Novel Method to Achieve Precision and Reproducibility in Exposure Parameters for Low-Frequency Pulsed Magnetic Fields in Human Cell Cultures

**DOI:** 10.3390/bioengineering9100595

**Published:** 2022-10-21

**Authors:** Michael Ronniger, Blanche Aguida, Christina Stacke, Yangmengfan Chen, Sabrina Ehnert, Niklas Erdmann, Georg Eschenburg, Karsten Falldorf, Marootpong Pooam, Anthony Wing, Margaret Ahmad

**Affiliations:** 1 Sachtleben GmbH, 20251 Hamburg, Germany; 2 Photobiology Research Group, Sorbonne Université CNRS, 75005 Paris, France; 3 Department of Biology, Faculty of Science, Naresuan University, Phitsanulok 65000, Thailand; 4 Siegfried Weller Institute for Trauma Research, Department of Trauma and Reconstructive Surgery, BG Unfallklinik Tübingen, Eberhard Karls Universität Tübingen, 72076 Tübingen, Germany

**Keywords:** HEK-Blue electromagnetic field, extremely low-frequency, EMF, PEMF, ELF-MF, inflammation, HEK-Blue, TLR4

## Abstract

The effects of extremely low-frequency electromagnetic field (ELF-MF) exposure on living systems have been widely studied at the fundamental level and also claimed as beneficial for the treatment of diseases for over 50 years. However, the underlying mechanisms and cellular targets of ELF-MF exposure remain poorly understood and the field has been plagued with controversy stemming from an endemic lack of reproducibility of published findings. To address this problem, we here demonstrate a technically simple and reproducible EMF exposure protocol to achieve a standardized experimental approach which can be readily adopted in any lab. As an assay system, we chose a commercially available inflammatory model human cell line; its response to magnetic fields involves changes in gene expression which can be monitored by a simple colorimetric reporter gene assay. The cells were seeded and cultured in microplates and inserted into a custom-built, semi-automated incubation and exposure system which accurately controls the incubation (temperature, humidity, ${\mathrm{CO}}_{2}$) and magnetic-field exposure conditions. A specific alternating magnetic field (<1.0% spatial variance) including far-field reduction provided defined exposure conditions at the position of each well of the microplate. To avoid artifacts, all environmental and magnetic-field exposure parameters were logged in real time throughout the duration of the experiment. Under these extensively controlled conditions, the effect of the magnetic field on the cell cultures as assayed by the standardized operating procedure was highly reproducible between experiments. As we could fully define the characteristics (frequency, intensity, duration) of the pulsed magnetic field signals at the position of the sample well, we were, for the first time, able to accurately determine the effect of changing single ELF-MF parameters such as signal shape, frequency, intensity and duty cycle on the biological response. One signal in particular (10 Hz, 50% duty cycle, rectangular, bipolar, 39.6μT) provided a significant reduction in cytokine reporter gene expression by 37% in our model cell culture line. In sum, the accuracy, environmental control and data-logging capacity of the semi-automated exposure system should greatly facilitate research into fundamental cellular response mechanisms and achieve the consistency necessary to bring ELF-MF/PEMF research results into the scientific mainstream.

## 1. Introduction

The biological effects of extremely low-frequency magnetic fields (ELF-MF) on living systems, occurring in the 5–300 Hz range and at intensities in the vicinity of the earth’s geomagnetic field, have received considerable attention in the past century [1,2]. On the one hand, safety and exposure limitations needed to be defined for ELF-MFs caused by man-made electromagnetic noise, which has led to regulatory statutes that continue to evolve [3]. On the other hand, the application of so-called pulsed electromagnetic fields (PEMF) has been empirically demonstrated to be an effective treatment for diseases such as arthritis [4,5,6,7,8,9], chronic pain [7,8,10,11,12,13], bone injury [14,15,16,17,18,19,20,21], wound healing [22] and hyper-inflammatory conditions [23,24]. This has resulted in publications on ELF-MF exposure of whole organisms as well as cells in culture, demonstrating effects on gene expression, cellular reactive oxygen concentration [25,26,27,28,29,30,31,32], membrane and lipid (raft) composition [33,34], ion channel activation [35] and literally dozens of other biological markers in differing cell types, organisms and plants [36]. A number of underlying mechanisms have been proposed for these effects, including the action of eddy currents induced by magnetic-field pulses [37]; ion cyclotron, stochastic [38,39] and paramagnetic resonance [40,41]; interference of quantum states of ions [42]; spin chemical effects on biochemical reaction intermediates [43,44,45]; magnetite nanoparticles [46]; and many more; see, e.g., discussion in [47]. However, despite a consensus that exposure to ELF-MF or pulsed electromagnetic fields (PEMF) indeed causes measurable biological effects in mammalian cell cultures, neither the exposure parameters required to elicit a defined response nor a coherent proven mechanism have been conclusively understood. As seen in the meta-analysis from Golbach et al. [48], numerous factors including differences in assay type, magnetic-field intensity, frequency, duration and cell type can lead to conflicting results.

As a result, one of the main difficulties hindering progress in the field has been the low reproducibility of experimental results from different labs in general [49,50], but specifically in the area of (electro-)magnetic field research. Poorly controlled biological processes, e.g., cell growth, in combination with the technical difficulties of defined magnetic-field exposure contributes to differing results among researchers [48]. Furthermore, as primary effects of these magnetic fields on biological systems are predicted to be weak, e.g., kT-problem discussion [47], even small deviations in temperature, humidity, lighting, or physiological or genetic differences in test materials from different sources can skew the results [51,52]. Furthermore, in most biological labs, the ambient electromagnetic conditions are not controlled. These vary considerably due to electric and magnetic noise produced by devices (e.g., incubators, smartphones, Wi-Fi, centrifuges, etc.), which are turned on and off unpredictably, as well as by distortions of the local geomagnetic field in the interior of a building. This problem is compounded by the fact that cell cultures placed inside commercial laboratory incubators are exposed to parasitic electric and magnetic fields (ELF-EMF) for the overwhelming part of the duration of an experiment. The intensity of these parasitic fields depends strongly on the incubator design and materials [52]. Sources of such fields could be the incubator heating system, internal pumps, fans and electronics. Finally, there is great inhomogeneity in magnetic-field exposure systems/devices themselves. In the case of commercially available PEMF devices, for instance, variables such as the signal amplitude, frequency, shape, slope and duty cycle have often been chosen using theoretical or observational considerations (e.g., Cnp pulse [53], the pulse used in [14] or the CIT-pulse (CIT, cell information therapy) used in [54]), but not critically examined to determine what component in the signal gives the biological effect. Yet all these variables can have effects on the experimental outcomes. As previously reported, the actual exposure received at the sample position is often not properly reported so that cellular exposure conditions are impossible to know, let alone replicate [55]. To tackle a substantial number of these technical hurdles to standardized incubation conditions using a reliable magnetic-field response assay, we describe the design of a dedicated magnetic-field exposure system for controlled ELF-MF application. The exposure system is used together with a standard operating procedure in the microplates format where all exposure parameters can be evaluated at the position of the samples and in real time (except for constant background fields) throughout the course of the experiment. To ensure a valid biological readout, the experiment uses a homogeneous, immortalized and genetically defined human reporter gene cell line (HEK-Blue™ hTLR4). This model cell line is used for studies of inflammation and is engineered to secrete alkaline phosphatase into the cell culture medium upon triggering the TLR4-dependent inflammatory response. As a consequence, using this experimental system, the biological readout is a simple colorimetric substrate assay, which can be readily standardized and replicated under laboratory conditions.

## 2. Materials and Methods

### 2.1. The Magnetic-Field Exposure System

The exposure system allows the application of controlled magnetic-field parameters with respect to signal shapes g(t), frequency *f*, intensity/amplitude $B_{0}$, duty cycle *D* as well as controlled environmental conditions characterized by temperature *T*, relative humidity rH and ${\mathrm{CO}}_{2}$ for the incubation of the cell culture. For each experiment, there are 2 parallel running systems: an ’exposure’ cell-culture incubator (called incubator E; IC E) to which the controlled magnetic field is applied and a ’control’ cell-culture incubator (called incubator C; IC C) running in parallel with no exposure condition. Both systems are controlled by an in-house developed software application.

#### 2.1.1. Incubator Design

The incubator housing is made out of black acrylic glass (PMMA) with an inner volume of 200 mm × 200 mm × 150 mm (x×y×z) enclosed by a water jacket to regulate the temperature via an external thermostat. A water bath provides passive humidity regulation. For faster temperature regulation, a Pt100-sensor (special design, Innovative Sensor Technology IST AG, ∅2.8 mm, 4-wire, quality class AA or F 0.1) connected to the thermostat is embedded directly in the water jacket. In addition, the incubator lid is temperature-controlled by the water circuit. An external ${\mathrm{CO}}_{2}$ controller provides a premixed ${\mathrm{CO}}_{2}$–air mixture. The gas mixture passes a humidifier (bubble water bottle) before entering the incubator. Since ${\mathrm{CO}}_{2}$ has a higher density than air, the incubator was constructed as a top loader. During an experiment, the internal incubator conditions regarding temperature, relative humidity and ${\mathrm{CO}}_{2}$ are logged by sensors. For temperature, an NTC sensor (negative temperature coefficient thermistor) is used and for humidity measurements a multisensor module (Ahlborn GmbH GmbH, Holzkirchen, Germany). The NTC sensor (TS-NTC-103A, B&B Sensors, Donaueschingen, Germany) was calibrated with a calibration bath (FK31-SL, Julabo GmbH, Seelbach, Germany) versus a DAkkS-calibrated Ahlborn Mess- und Regelungstechnik GmbH, Eichenfeldstraße 1, 83607 Holzkirchen, Germany (Supplement, Calibration Certificate 2019-06)) Pt100 (PT100 1/10th DIN liquid probe, ∅4.8 mm × 40 mm long, Electronic Temperature Instruments Ltd. (ETI), Easting Close, Worthing, Sussex, BN14 8HQ, UK) with an absolute error of ΔT=±0.1 K (Supplement, Figure S7). The NTC sensor and the multisensor module were placed within the coil in vicinity of the sample and close to the inner wall of the coil. The precision of the atmospheric sensors in the incubator are ΔT=0.1 K, ΔrH=0.1% and $\Delta [{\mathrm{CO}}_{2}]=0.1\%$. For ${\mathrm{CO}}_{2}$ measurement, we use a heated flow through IR-${\mathrm{CO}}_{2}$-sensor (SPRINTIR-WF-20, Gas Sensing Solutions Ltd., Cumbernauld, UK) logging the incubator gas. During the experiments, the setpoints for temperature and ${\mathrm{CO}}_{2}$ in the incubators are: 37 °C, 5%${\mathrm{CO}}_{2}$, while rH is passively regulated by evaporation.

#### 2.1.2. Coil Specifications

**Figure 1:**
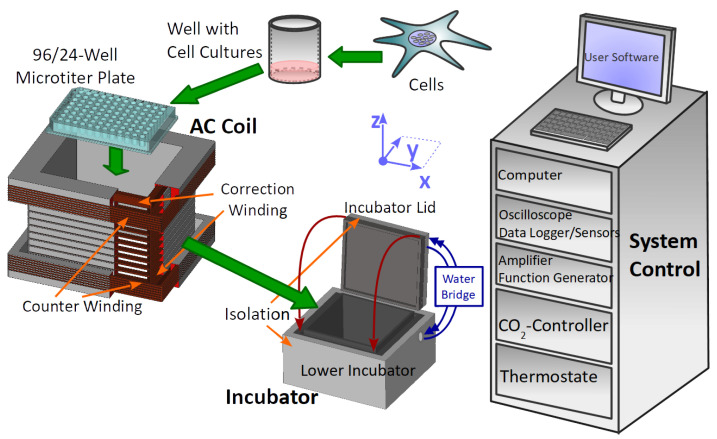
Experimental setup containing the biological sample in the microplate. The cell-culture plate fits into the rectangular coil which generates the AC magnetic field. Both culture plate and coil are placed in the incubator regulated by thermostat and ${\mathrm{CO}}_{2}$-controller. Atmospheric conditions in the incubator are logged with sensors. Function generator and amplifier generate the current for the coil. All active devices are connected and controlled by the system control computer, which provides a user interface to manage experimental settings and triggers the EMF exposure automatically. The inner part of the incubator housing (lower incubator and incubator lid) is surrounded by a styropor thermal isolation. The bottom part and the heated lid are connected via a water bridge. The coil has an inner winding including the correction winding and an outer part with a counter winding for far-field reduction. An oscilloscope allows the indirect measurement of the AC magnetic field by using a shunt and the B/I calibration of the coil. A summary of all devices is given in the Supplement, Table S1.

The biological sample is placed into a plexiglass holder at a fixed position inside the coil, which is then placed in the incubator. The coil was constructed to provide a homogenous field distribution regarding field intensity across the entire microplate (Supplement, Figure S1). The homogeneity is within ≤1% over a space of 3 stacked microplates. Furthermore, the coil reduces the far field (via a counter winding) to avoid the exposure of nearby biological samples such as the control samples. A distance of ≳2 m between the control and exposed samples reduces the cross-contamination to by a factor of $10^{6}$ in intensity (Supplement, Figure S2). Magnetic-field calculations were performed analytically by using Biot-Savart law for rectangular geometries. In addition to a precise magnetic-field distribution, the coil has a wide frequency response spectrum, which allows the application of magnetic pulses in the μs-range (Supplement, Figure S4). Figure 1 shows the structure of the coil including the wiring. There are 3 types of windings implemented in the coil: a winding to produce the major part of the center field, a correction winding to increase the field homogeneity in the inner volume and a counter winding to reduce far field effects. PVC isolated flexible wire (∅2.1 mm) was used to reduce the parasitic capacitance by increasing the inter-wire distance to increase the usable frequency range. Furthermore, the coil was double-wrapped with wire (LiY 1.0 mm^2^, ∅2.1 mm) to avoid an electric field generation between the electric input and output. The technical parameters of the coil are summarized in Table 1.

#### 2.1.3. Magnetic-Field Signal Generation

**Figure 2:**
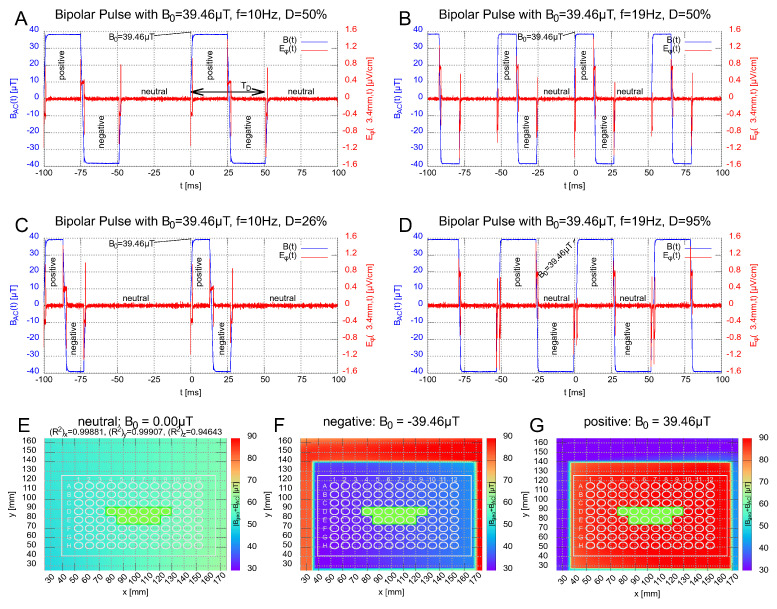
Subfigures (**A**–**D**): Show the signal shape of the magnetic field (blue) for the bipolar rectangular pulses with 10 Hz and 19 Hz, respectively. The altered magnetic-field duration $T_{D}$ (defined in subfigure (**A**)) was chosen to be $T_{D}=26.32$ ms and $T_{D}=50$ ms. Each bipolar signal has three states, labeled with *positive*, *negative* and *neutral*. The duty cycles of the signal in (**C**,**D**) were chosen to reflect a $T_{D}$ equivalent to the signal in (**A**,**B**), respectively. The red curve represents the Φ-component of the induced electric field $E_{\Phi }$, which was calculated by the induction law (Supplement, Section S3.3) and stays below ≤$1.6\times 10^{-6}\;\frac{V}{m}$ for all fields at well boundary at R=3.4 mm (∅6.8 mm). Subfigures (**E**–**G**): Show the field distributions of the total magnetic field (Supplement, Section S3.2 for definition) for the three states. The wells used for the samples are labeled in green.

All magnetic signals are generated by an arbitrary function generator (33511B, Keysight Technologies Inc., 1400 Fountaingrove Parkway, Mailstop 1 USM, Santa Rosa, CA 5403, USA) connected to the 4-quadrant amplifier (DCP 130/60 HSR, Servowatt GmbH, 70839 Gerlingen, Germany) and custom-made coil (Sachtleben GmbH, 20251 Martinistraße 64, Hamburg, Germany). The amplifier is used as a current regulator in order to provide an electric current proportional to the input voltage. This avoids a temperature-induced voltage drift caused by varying coil resistance due to temperature differences in the incubator. The function generator–amplifier–coil setup needs to be frequency and amplitude calibrated. The measured frequency and phase responses are shown in the Supplement, Figure S5. Calibration allows the input signal to be tuned so that the output magnetic field fits the desired signal shape. This pre-distortion of the input signal is calculated as described in the Supplement, Section S2.4. This procedure enables the application of very short pulses beyond the amplifier range of 200 kHz. In our current benchmark experiment, we generated rectangular signals, which were measured with a differential oscilloscope (PicoScope 4444, Pico Technology, St. Neots Cambridgeshire, United Kingdom) during the entire course of the experiment. A cutout of these signals is shown in Figure 2A–D.

#### 2.1.4. Software

All devices used in the exposure system are logged and controlled by a custom-designed software program, which allows easy adjustment of the experimental parameters including scheduling of the magnetic-field exposure, the logging of sensor data as well as the operating state of all devices. At any time, the user has the ability to check the atmospheric conditions within the incubator, the lid status (open/closed) and the status of the exposure itself. All measured values inside the incubator are automatically controlled and logged for the duration of the entire experiment.

Additionally, each log file is stored and shared via cloud backup for parallel and independent analysis. A technical team can monitor each session in real time and is able to remotely control the program in case of anomalies or adjust new experimental settings.

### 2.2. Magnetic-Field Exposure Conditions

The magnetic field generated by the coil ${\vec{B}}_{AC}\left(\vec{r}\right)$ and the local geomagnetic field ${\vec{B}}_{geo}\left(\vec{r}\right)$ interfere with each other and provide different magnetic-field conditions at the sample positions within the incubators. They can be characterized by the following parameters, summarized in Table 2.

We chose 4 bipolar rectangular signals comprising 3 different magnetic field states, ’positive’, ’negative’ and ’neutral’ (zero). They are shown and labeled in Figure 2A–D. The corresponding field distributions of the (absolute) total magnetic field (superposition of the alternating and geomagnetic field; Supplement, Equation S3 for definition) for the 3 states are shown in Figure 2E–G. Inspired by the 10 Hz PEMF signal employed in [24], which showed a decrease in the inflammatory response in HEK-Blue™ hTLR4 cells for pulsed electromagnetic fields, a frequency of f=10 Hz was chosen. Additionally, as prior studies showed effects of low-level static magnetic fields on the inflammatory response [24], we chose an amplitude of $B_{0}=$ 39.46 μT, which transiently decreases the *z*-component of the total magnetic field when the pulsed field is directionally opposite to the geomagnetic fields, while the $B_{x}$ and $B_{y}$ components remain as the residual field, see Figure 2F. In further experiments, we investigated the effects of changing the frequency and the altered magnetic-field duration $T_{D}$. The latter is defined as the duration of the pulse with non-zero magnetic field. A graphical definition is provided in Figure 2A (double-headed arrow). The altered magnetic field duration is directly connected to the frequency and duty cycle by

(1) $$T_{D}=\frac{D}{f}=D\;T$$

with $f:=\frac{1}{T}$. In total, 2 frequencies (f=10 Hz and f=19 Hz) and 2 altered magnetic-field duration conditions ($T_{D}=26.32$ ms and $T_{D}=50$ ms) were tested for the rectangular signal, Figure 2A–D. Finally, the duty cycle was chosen to be D=50% for the signals in Figure 2A,B. For Figure 2C,D, the duty cycle has been adjusted so that $T_{D}$ equals that of signal B and A, respectively, see Table 2. In addition, we used two PEMF signals with a proprietary, monopolar, triangular shape and a short pulse length $T_{D}\leq 100\;\mu$s (so-called CIT-pulse [21,56]).

In Figure 2A–D, signals were measured with the oscilloscope (PicoScope 4444) via a shunt resistor $R_{S}=0.2\;\Omega$ (part of the amplifier DCP 130/60 HSR) and re-scaled to proportional magnetic field via the B/I-calibration slope $K=150\;\frac{\mu T}{A}$ in the center of the coil. The maximum induced electric field $E_{\Phi }$, which represents the Φ-component in cylindrical coordinates within a microplate well (∅6.8 mm), was estimated to be ≤1.6 $\times 10^{-6}\;\frac{V}{m}$ (Supplement, Equation S4, Section S3.3). This is very low compared to the cell-membrane electric field ≥4.0 $\times 10^{6}\;\frac{V}{m}$, which was estimated from the membrane potential in the range of −80 mV to −40 mV for a membrane thickness of 5–10 nm. The other components, $E_{\rho }$ and $E_{z}$, vanish due to cylindrical symmetry. The local constant geomagnetic field within the incubators were measured with a customized travel drive apparatus using a 3-axis magnetic probe (RM3100, PNI, 2331 Circadian Way, Santa Rosa, CA 95407, USA) and vary by roughly $\Delta |\vec{B}|=\pm 5.0\;\mu$T over the length of the well plate and by $\Delta |\vec{B}|=\pm 2.5\;\mu$T over the wells used marked green in Figure 2E–G.

### 2.3. The Biological Reporter System

#### 2.3.1. Cell-Culture Conditions

**Figure 3:**
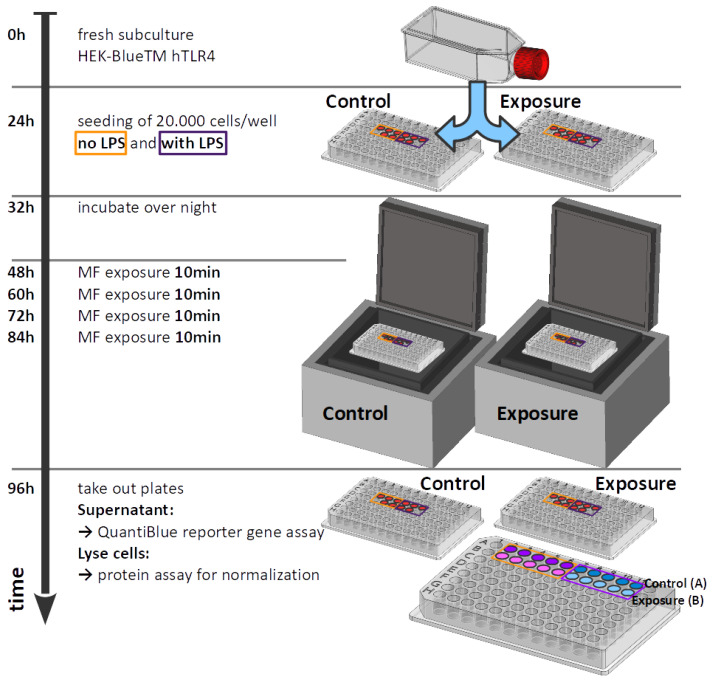
Time sequence of the experiment.

Cell-culture conditions are taken from reference [24] and were performed as follows: Human embryonic kidney HEK293 cell lines stably expressing human TLR4 (#hkb-htlr4; InvivoGen, San Diego, CA, USA, June 2000, https://www.invivogen.com/hek-blue-htlr4, accessed on 28 July 2022) were used for all experiments. HEK-Blue™ hTLR4 cells express an alkaline phosphatase (AP) reporter gene regulated by NF-κB and AP1 transcription factors. The quantification of cell infection was measured by assaying alkaline phosphatase activity in cell-culture medium containing colorimetric enzyme substrates. Cells were cultured in DMEM high glucose (Sigma, St Louis, MO, USA) containing $4500\;\frac{\mathrm{mg}}{L}$ of glucose, 10% (*v*/*v*) heat-inactivated fetal bovine serum (Gibco, Dublin, Ireland) and 1×HEK-Blue Selection solution (InvivoGen, San Diego, CA, USA) and grown at 37 °C under a humidified atmosphere at 5% ${\mathrm{CO}}_{2}$ in a dedicated incubator (MCO-18AC, Panasonic Biomedical, Leicestershire, UK). Cells were first amplified in 75 mL culture flasks and sub-cultured every 72 h. For experimental trials, HEK-Blue™ hTLR4 cells were seeded from a single stock culture flask at a density of 20,000 cells per well in 96-well plates. Inflammatory response was stimulated at seeding by incubation with bacterial lipopolysaccharide (LPS) dissolved in phosphate buffered saline (PBS) (Sigma, MO, USA). A final concentration of $100\;\frac{\mathrm{ng}}{\mathrm{mL}}$ LPS was used for all tests. Negative control cultures were obtained by adding PBS at the same volume as the LPS substrate to the control culture medium. LPS serves as positive control on every assay control plate. After LPS addition, cell cultures were incubated for a further 16 h before transfer to the relevant exposure incubator (ELF-MF/PEMF exposure). All cultures were grown in parallel from the same cell stock culture and under identical conditions. Figure 3 shows an overview of the time sequence of the experiment.

By placing the microplates within the plastic incubator (Figure 1), the experiment was started and lasted 48 h with no intermediate incubator opening (for medium or reactions components change). Five wells were seeded as replicates in a single experiment, using a 96-well format plate. All of the samples were placed in the center portion of the plate, far from the borders where inhomogeneities might occur (see Figure 2E–G for sample positions). ELF-MF/PEMF exposure conditions were as described (see Table 2).

#### 2.3.2. Alkaline Phosphatase Assay for Monitoring Inflammation

The inflammatory response of HEK-Blue™ hTLR4 cells was measured by determining the enzyme activity of the secreted alkaline phosphatase (SEAP) reporter gene, which was normalized to the total concentration of cells per well. SEAP enzyme activity was assayed at the end of the 48 h growth period by removing the equivalent of 7μL of cell-free supernatants from each of five duplicate wells subjected to the treatment condition. The culture media samples were then mixed with 180μL of QUANTI-Blue™ detection solution (Invivogen), which contains the AP colorimetric substrate, and incubated in accordance with manufacturer’s specifications at 37 °C, 5% ${\mathrm{CO}}_{2}$ for 20 min in a fresh 96-well plate. Alkaline phosphatase activity was measured as the absorbance of the detection solution at 620 nm using an Epoch microplate reader (BioTek, Winooski, VT, USA). Values from five duplicate wells were averaged to obtain a single experimental data point. In order to detect possible differential cell growth effects resulting from these treatments, the HEK-Blue™ hTLR4 cells were also measured for total protein concentration in each well after the treatment period, using the DC Protein Assay kit (Bio-Rad Laboratories, Mississauga, ON, Canada). Briefly, the culture medium was removed from each of the n=5 duplicate wells subjected to experimental conditions. A total of 30μL of cell lysis buffer (25 mM Tris-HCl pH 7.4, 150 mM NaCl, 1% NP-40, 1 mM EDTA, 5% glycerol) was added to the cells inside the culture wells and incubated for 1 h at 4 °C to induce cell lysis and achieve protein solubilization. Then, 15μL of lysate was transferred into a fresh 96-well plate and mixed with the DC protein assay reagents as recommended by the manufacturer. The levels of total proteins were measured by absorbance at 750 nm by an Epoch microplate reader (BioTek). The absorbance value of QUANTI-Blue™ Solution (*OD*620), representing secreted alkaline phosphatase activity, was subsequently normalized to the total protein concentration (*OD*750) and presented as a ratio (*OD*620/*OD*750) defined by

(2) $$Q:=\frac{OD^{\left(620\right)}}{OD^{\left(750\right)}},$$

A background level of alkaline phosphatase secretion was observed in cell cultures that had not been exposed to LPS after the 48-h incubation period and, therefore, did not respond to the inflammatory treatment. This background SEAP value was subtracted from the values obtained from the LPS-stimulated cell cultures to obtain the TLR-4 dependent component of the inflammatory response. Data were normalized to non-exposed LPS-treated cells and, finally, the effect of treatment is expressed as the percentage of inflammation achieved after LPS induction in ELF-MF/PEMF-treated groups as compared to the SEAP secretion response of control cells that had received LPS stimulation without additional ELF-MF/PEMF treatment. The inflammatory response is defined by:

(3) $$R_{IR}:=\frac{\bar{\Delta Q_{E}}}{\bar{\Delta Q_{C}}}$$

The raw data for Equation (2) and further definitions are found in the Excel file “Raw-data.xlsx” and Supplement Section S5.1.

### 2.4. Statistical Analysis

All data were analyzed by using GraphPad Prism version 7.4.2 for Mac (GraphPad Software, La Jolla, CA, USA). Data were analyzed for normality with the Shapiro–Wilk test. Results are expressed as mean ± standard deviation (SD), including propagation of errors. The difference between treated and control conditions for the inflammatory response were compared using one-way ANOVA followed by the Dunnett’s test and Cohen’s *d*-test, see Table S4. Between 3 and 8 independent experiments were performed at each condition. Differences were considered statistically significant with a *p*-value <0.05 (*), <0.01 (**), <0.001 (***), <0.0001 (****). Cohan’s *d*-test was performed with effect size evaluation of Bravais–Pearson correlation coefficient *r*-value ≤0.1 weak, ≤0.3 medium and ≥0.5 strong effect.

## 3. Results

### 3.1. Technical Experimental Setup

The setup consists of a custom-built magnetically transparent incubator with a semi-automated exposure system for magnetic fields in a modular setup. The magnetic-field exposure conditions are generated by a square coil designed to provide a uniform distributed signal to all wells in a microplate and can be precisely tuned to the frequency, signal shape, amplitude, treatment duration and number of treatments per day through a computer-controlled program. Before starting the experiments, the AC magnetic-field exposure was assessed at the position of the microplate by the electric current and the well-known field geometry of the coil. We generated six different signal shapes for the biological experiments: four rectangular (Figure 2A–D) and two proprietary triangular short pulses (not shown). The geomagnetic background field was measured after installing the system in the lab and the measurements were repeated in certain intervals at a specific position in the coil. No mentionable drifts were detected. Hence, the magnetic-field exposure conditions remained the same at each well position on a microplate (Figure 2E–G).

**Figure 4:**
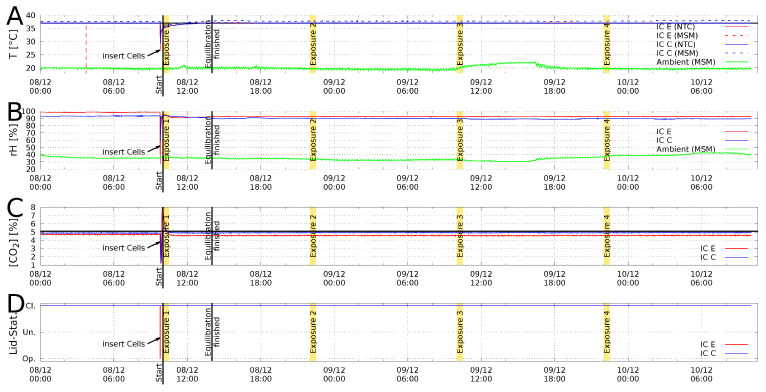
Example for a log file plot from Incubator C (blue curves) and E (red curves). (**A**): Temperature T in [°C]; (**B**): relative humidity rH in %; (**C**): ${\mathrm{CO}}_{2}$ concentration [CO${}_{2}]$ in %; (**D**): Lid status open/undefined/closed. Exposure durations are marked yellow and the ambient temperatures are represented by green lines. Double temperature measurement with negative temperature coefficient NTC sensor (solid) and a multisensor module (MSM, dashed).

As a feature of the live monitoring of the experiment, we can see that the control and exposed microplates experienced the same atmospheric conditions throughout the whole experiment (Figure 4).

### 3.2. Effects of Pulsed Magnetic Field on the Inflammatory Response

In order to demonstrate the effectiveness of the magnetic-field exposure system, we present the effect of applied magnetic fields on the TLR4-dependent inflammatory response pathway, which can be studied in an engineered cell-culture system (HEK-Blue™ hTLR4). When inflammation is induced via artificial means, e.g., a bacterial elicitor such as Lipopolysaccharide (LPS), the resulting increase in alkaline phosphatase secretion can be measured by a simple colorimetric assay. Thus, any change in the inflammatory response due to the magnetic field is determined by a colorimetric assay subsequent to exposure.

**Figure 5:**
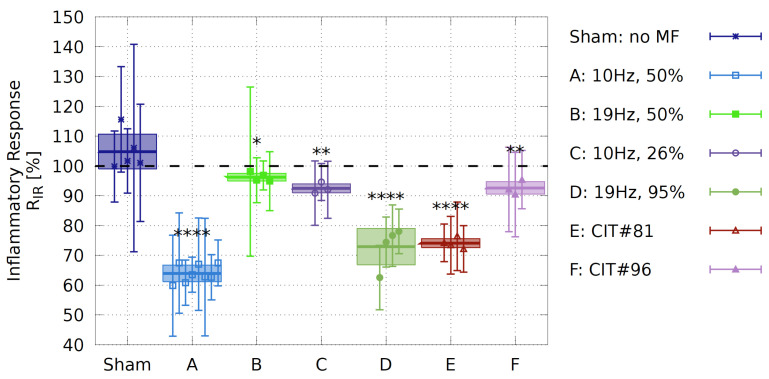
Effect of magnetic-field exposure on inflammatory response in HEK-Blue™ hTLR4 cell cultures. Each data point (i) is represented by the ratio $R_{IF}^{\left(i\right)}$ in the inflammatory responses (Supplement, Equation S8a). Box plots represent the mean over the biological repeats ± standard deviation. Sham experiment: experiments were conducted without magnetic-field exposure in both of the incubators and served as a measurement of the background variation between identical experiments. MF-exposure conditions are described in Section 2.2 and the data is shown in the Supplement, Tables S3 and S4. N: number of biological repeats (Sham N=5, A N=8, B N=3, C N=4, D N=4, E N=4, F N=3); p<0.0001 ****, p<0.01 **; p<0.05 * represent *p*-value significance in comparison to sham exposure; the correlation coefficient of Cohan’s *d* test is also shown in the Supplement, Table S4 quantifying a strong effect size (r>0.5).

To ensure that no exposure parameters in the room beyond our control could influence the experimental outcome, a series of sham experiments were conducted. Neither the “exposed” nor the “control” incubators provided any ELF-MF/PEMF stimulation. Of n=5 independent experiments, performed at different weeks with different cell passages, there was no significant variation in the inflammatory response between the two incubators (see Figure 5, “Sham”). These data indicated that there was no significant background variation between incubators during an experiment and provided a baseline for all subsequent experimental results (Methods: see statistical analysis Section 2.4 and Supplement, Section S5.1).

A prior study reported that a 10 Hz pulsed magnetic-field signal from a commercially available device could elicit a decrease in the inflammatory result in HEK-Blue™ hTLR4 cells after only two days of exposure treatments [24]. In an effort to replicate these findings under more standardized conditions, we used a defined pulse condition (Signal A, 10 Hz frequency, 50% duty cycle, rectangular, bipolar, 39.6μT amplitude, Figure 2A). This signal was applied to cell cultures for ten minutes, once every twelve hours, over two days of growth. At the end of this time, we assayed for the inflammatory response by colorimetric assay, and observed a decrease by almost 37% in inflammatory response as compared to cell cultures in the *control* incubator (Figure 5, Signal A). The inflammatory inhibition was significantly higher than the decrease in inflammation reported in [24], indicating that our specific 10 Hz signal was more effective.

### 3.3. Effect of Varying the Altered Magnetic-Field Duration $T_{D}$ and Frequency f on the Inflammatory Response

Here, we take advantage of the unique tunability and precision of the experimental setup to dissect the active parameters within an ELF-MF/PEMF experiment, by changing precisely just one parameter at a time. Starting with changing the frequency on our HEK-Blue™ hTLR4 cell cultures and whether the anti-inflammatory effects could be further optimized. All rectangular signals are presented in detail in Figure 2A–D and Table 2. Changing the frequency from 10 Hz (Signal A) to 19 Hz (Signal B) with the same duty cycle (see Equation (1)) of 50% led to the result that cells did not respond to the 19 Hz (see Figure 5, Signal B). Therefore, it appeared that the 10 Hz, but not the 19 Hz, frequency, was effective in eliciting a biological response and that we were able to isolate this parameter. However, by changing the frequency from 10 Hz to 19 Hz, the altered magnetic-field duration $T_{D}$ is reduced. We, therefore, tested the effect of a changed $T_{D}$ whilst keeping the 10 Hz exposure frequency by decreasing the duty cycle from 50% to 26%, resulting in a decrease in altered magnetic-field duration from $T_{D}=50$ ms to $T_{D}=26.32$ ms. Under these conditions, the 10 Hz frequency was no longer as effective in promoting an anti-inflammatory effect (Figure 5, Signal C). Thus, for the rectangular pulses used, besides the frequency, the altered magnetic-field duration seems to be an important parameter.

Last, we used the 19 Hz frequency and increased the duty cycle from 50% to 95% (Signal D, see Figure 2D), resulting in a longer altered magnetic-field duration from $T_{D}=26.32$ ms to $T_{D}=50$ ms, comparable to that under Signal A (10 Hz, 50% duty cycle). In this case, the 19 Hz signal indeed induced a significant reduction in the inflammatory response by roughly 27% and the biological activity of the Signal A was restored (Figure 5, Signal D). To our knowledge, this was the first time that such a biological effect was restored by changing just one parameter.

### 3.4. Effect of Short-Pulsed Magnetic-Field Signals

As a further verification of the ability of our system to provide biologically relevant and well-controlled exposures to cell cultures, we tested two additional short-pulsed signals E and F (CIT#81 and CIT#96), which were featured in studies of [54,56]. With Signal E (Figure 5E, CIT#81), we showed that a signal with $T_{D}$ in the μs-range ($T_{D}<100\;\mu$s) is also able to significantly decrease the inflammatory response in HEK-Blue™ hTLR4 cells by roughly 25%. However, a similar μs-pulsed signal gave no biological effect (Figure 5F, CIT#96). Although the two CIT-signals E and F differ only by Δf≈0.5 Hz in their frequency, they differ significantly in their on–off burst patterns, suggesting additional parameters to be evaluated in the future for the optimization of pulsed fields.

## 4. Discussion

In order to avoid the many uncertainties inherent in the field of biological magnetic-field effects, in recent decades a number of technical EMF-exposure setups were developed [57,58,59,60] allowing more precise control of magnetic-field exposure according to their intended purpose. Furthermore, technical guidelines have been published to standardize the exposure of magnetic fields and describe important issues in bioelectromagnetic research [61]. However, the reproducibility of EMF cell experiments remained a challenge with many systems and technical setups being used by numerous research groups in this field [62].

In this work, we have approached the above-mentioned obstacles in a holistic manner by considering biological and technical aspects with equal importance to reduce errors and ensure the reproducibility of EMF cell experiments by the control of major conditions.

### 4.1. Technical System

The advantages of the technical system can be summarized by the following points:

- Flexible, modular design;
- Automated control and logging of exposure and incubation conditions;
- Same setup and handling for control vs. exposed condition;
- Reduction of noisy ELF-magnetic background fields during incubation;
- Ability to manipulate well-known magnetic field parameters;
- Continuous technical remote support and monitoring.

Compared to other systems [57,58,59,60], the new set up presented here allows a user-friendly adjustment of various magnetic-field parameters by different coils and signal shapes. It automates the logging of environmental parameters, such as temperature, humidity, ${\mathrm{CO}}_{2}$ concentration and signal amplitude throughout the course of the experiment and collects those data to provide a comprehensive set of parameters for each experiment, as shown in Figure 4. This helps and simplifies the detection of inconsistencies and errors during the experiment. Potential ELF-MF background effects (as in approaches involving commercial incubators) are reduced by using a dedicated plastic incubator for each sample. A high homogeneity (Supplement, ±1%, Figure S1C,D) of the alternating field allows to expose the samples equally across the section of the well plate used. Nevertheless, local homogeneities in the geomagnetic background field are not shielded and can influence the cells, as shown in Figure 2E–G. The local spatial deviation in an incubator was estimated to be between ±2.5μT for the samples.

Fluctuations in temperature, relative humidity and ${\mathrm{CO}}_{2}$ concentration for incubator E and C can be calculated from the log files (Figure 4), which are characterized by the mean, standard deviation, minimum and maximum values (Supplement, Table S2). In order to estimate the system’s accuracy, the time after reaching equilibrium condition until the termination of the experiment was used to extract the above-mentioned values. The minimum and maximum values for the temperature differ only by $T_{min}-T_{max}=0.19$ K for incubator E and $T_{min}-T_{max}=0.23$ K for incubator C. This also includes the ohmic heating during the exposure and ambient/room temperature fluctuations. The passive controlled humidity remains in the range $rH_{min}-rH_{max}=0.70\%$ for incubator E and $rH_{min}-rH_{max}=2.50\%$ for incubator C, respectively. Similar for ${\mathrm{CO}}_{2}$ concentration, [CO${}_{2}{]}_{min}-{\left[{\mathrm{CO}}_{2}\right]}_{max}=0.15\%$ for incubator E and ${\left[{\mathrm{CO}}_{2}\right]}_{min}-{\left[{\mathrm{CO}}_{2}\right]}_{max}=0.12\%$ for incubator C, which is very close to the ${\mathrm{CO}}_{2}$-accuracy of the ${\mathrm{CO}}_{2}$-controller (0.1%). This accuracy is entirely consistent with commercial incubators as used, for instance, in [57].

### 4.2. Biological Assay

The HEK-Blue™ hTLR4 cell line serves as a widely used model system [63,64,65] for inducing and studying the TLR4-dependent inflammatory response, which provides a genetically controlled, homogeneous, readily available material with a readout that can be scored by a simple colorimetric assay. In addition, HEK-Blue™ hTLR4 cell lines fulfill the following key points as a biological marker necessary to demonstrate magneto-sensitive effects in in-vitro experiments:

- Low biological sample variation due to reporter cell line instead of primary cells;
- Suitable experimental procedure to see phenological markers within a short time period;
- Easy to handle, robust but highly sensitive assay.

Furthermore, excessive TLR4-dependent inflammatory response has been implicated in several severe clinical conditions including atherosclerosis, rheumatoid arthritis, neuroinflammation, and trauma and hemorrhage, as reviewed by [66] or more recently also in SARS-CoV-2-induced hyperinflammation [24,67]. The result of this study, using signal A causes roughly 37% (Figure 5A) inhibition of the inflammatory response, was entirely consistent with data from prior reports [24].

We observed that not only the signal frequency was leading to biological effects, but also the altered magnetic field duration $T_{D}$. For example, Signal A, with a 10 Hz frequency and $T_{D}=50$ ms, reduced the inflammatory response by 37%, whereas Signal C, also at 10 Hz, with a reduced $T_{D}=26.32$ ms, results in a less than 10% reduction. Similarly, Signal D at 19 Hz frequency and $T_{D}=50$ ms, was biologically effective (with an inflammatory response reduced by roughly 27%), whereas Signal B at 19 Hz frequency and smaller $T_{D}=26.32$ ms had much less effect on the inflammatory response (<10%). Effect sizes are summarized in Table S4 in the Supplement (column 3 for ${\bar{R}}_{IR}$). Therefore, besides frequency, some aspects of signal duration and/or shape have an impact on biological activity.

In the experiment, we aimed at a reasonable compromise between precision and feasibility in the biological and technical system. To the best of our current knowledge, we were able to identify frequency *f*, waveform g(t), pulse-burst pattern and the altered magnetic-field duration $T_{D}$ as influential parameters on an anti-inflammatory effect. Nevertheless, the influence of signal amplitude $B_{0}$ and the constant background magnetic field still needs to be investigated. The platform used here will serve for reproducing the results at a second site, as well as for further studies regarding additional parameters such as the impact of background fields [68] or amplitudes. The technical system and method presented here is very suitable to enable research of two critical questions in the future. First, to conduct screening cell experiments to identify parameters of high clinical relevance (e.g., using primary cells) and, second, to investigate the linkage between mechanisms and certain parameters to gain a deeper fundamental understanding of how EMF and biological cells interact.

### 4.3. Limitations of the Study

The empirical results reported herein should be considered in the light of some limitations. Firstly, all biological samples are subject to a certain level of variation. It is very difficult to achieve identical physiological responses in two different cellular cultures that have been independently passaged and grown from different parent stocks in different experiments at different times [49,50]. Therefore, instead of a time-consuming and costly maximum control of biological experimental conditions, we performed comparative experiments in this study. Given the homogeneity of the cells and standardized protocols applied to cell-culture and assay handling, the precision reached (sham–sham variability: 5%) is considered a reasonably achievable precision in terms of effort benefit ratio.

Furthermore, there is an ongoing debate concerning the importance of ‘double blinding’ in life science studies [69]. We have not blinded the experiments, because the focus of this proof-of-concept study was on optimization the interaction of the technical and biological systems. In the future, we can address blinding (blinding features are already integrated in the software) and changes to the biological processing and technical setup.

Finally, in this study only a selection of possible magnetic-field parameters was considered. The experiment was restricted to modifying those parameters known to have an effect, while keeping other parameters unchanged. However, the technical system was designed to examine all relevant magnetic-field parameters (frequency, duty-cycle, amplitude, pulse rise/fall time) and can be adjusted accordingly. Parameter studies will be the subject of further publications.

## 5. Conclusions

This paper describes the development and characteristics of a functional exposure and incubation system and its application for the control of ELF-MF and PEMF in biological samples. The potential to efficiently screen effective ELF-MF/PEMF parameters and resolve complex parameter setups to gain a better understanding of underlying mechanisms of magnetic field action on biological systems was demonstrated. In combination with a highly sensitive and well-established biological inflammatory response assay, it was possible to generate highly significant and reproducible magnetic-field effects of a 37% decline in a TLR4-dependent inflammatory response with a 10 Hz and 50% duty-cycle rectangular signal. For rectangular signals, we observed that not the frequency alone, but also the altered magnetic-field duration $T_{D}$, related to duty cycle and frequency by Equation (1), was responsible for the biological effect. A $T_{D}=50$ ms induces a significant anti-inflammatory effect in the range of 27–37%. A lowered $T_{D}$ reduced the effect size significantly below <10%. However, short-pulsed fields with triangular shape and $T_{D}\leq 100\;\mu s\ll 26.32$ ms could also induce significant anti-inflammatory effects of a similar order of magnitude to the rectangular signals. Thus, $T_{D}$ is not the only effective parameter. Possibly, different magneto-sensitive mechanisms could be involved on different time scales for $T_{D}$, which correspond to different frequency ranges due to the Fourier analysis of the magnetic signal. Obviously, more extensive magnetic-field parameter studies are necessary allowing clarification on a deeper level of understanding of the fundamental mechanisms.

These findings show that the HEK-Blue™ hTLR4 reporter cell line is an excellent model system for studying immune modulatory effects of ELF-MF/PEMF fields. The power of our custom-built exposure and incubation system for identifying and characterizing magnetic-field effects in living systems is demonstrated by the anti-inflammatory effects reproduced in a commercially available model cell-culture system.

Using this exposure and incubation system, it is possible to obtain reliable, accurate and fast information regarding the understanding of different parameters of ELF-MF effects in different types of human cell cultures. In particular, low-intensity exposure levels ($B_{0}=39.46\;\mu$T) with different signal shapes will be relevant for a wide audience of researchers. Such groups are hereby invited to participate in this endeavour to explore new therapy options. Relevant magnetic-field parameters can be further varied and magnetic field research can be accelerated with the system described. Looking forward, there is a smaller version of the system planned, which can be more easily adapted to established lab procedures and protocols.

In conclusion, the synthesis of a robust and sensitive biological readout system and a technically well-controlled environment demonstrate the potential to generate reproducible results in studying magneto reception and allow systematic exploration of the effect of critical ELF-MF/PEMF parameters for given cell models/systems. The experiments shown present proof of principle that the exposure and incubation system can be used both to develop future applications of magnetic-field therapy in medicine and to elucidate the underlying fundamental cellular response mechanisms triggered by magnetic fields. Given that this methodology is easily applicable to multiple cell types, this would mean a great leap forward in terms of reproducibility within the research field by installing identical systems in different laboratories, to investigate magneto-receptive effects by the application of nearly identical magnetic fields.

## Figures and Tables

**Table 1:** Technical parameters of the coil. ${}^{a}$ The B/I ratio was calibrated by measuring the coil current with a calibrated multimeter (Supplement, calibration certificate ‘Keithley DAQ6510’) versus the magnetic-field intensity (Supplement, Figure S3) with calibrated Fluxgate magnetometer (Supplement, calibration certificate for ‘FLUXMASTER’). ${}^{b}$ The inductance was measured by the frequency response in a LCR-resonant test circuit with a known capacitance.

Parameter	Symbol	Value
Outer dimensions	L×W×H	195 mm × 166 mm × 130 mm
Winding number	$N_{1}$ inner, $N_{2}$ correction, $N_{3}$ counter	26, 2 × 6, 2 × 7
B/I-ratio at the center ${}^{a}$	*K*	≈150$\frac{\mu T}{A}$
Ohmic resistance	*R*	0.8Ω
Inductance	*L*	≈0.235 mH ${}^{b}$
Wire	-	LiY 1.0 mm^2^, ∅2.1 mm

**Table 2:** Summary of the magnetic-field parameters. Frequency *f*, period *T*, duty cycle *D* and the altered magnetic-field duration $T_{D}$ related to the duty cycle by Equation (1). The definition of $T_{D}$ is shown in Figure 2A. The geomagnetic background field was measured with a 3-axis magnetic-field sensor positioned by a custom-made travel drive apparatus after building up the experimental setup in the lab. Here, we summarize characteristic values for the components and the total magnetic field at relevant well position, e.g., D5. Further information on the field distributions for both incubators are shown in the Supplement, Figures S5 and S6. In total, 6 signals were tested. They all have the same amplitude and the same geomagnetic background field homogeneity in both incubators (IC E and IC C). Signals A–D are rectangular while E and F are very short-pulsed and of triangular shape.

Parameter	Signal	A	B	C	D	E	F
Frequency	*f*	10 Hz	19 Hz	10 Hz	19 Hz	52.3 Hz	51.8 Hz
Period	*T*	100 ms	52.63 ms	100 ms	52.63 ms	19.12 ms	19.31 ms
Duty cycle	*D*	50% 50%	26%	95%	<0.523%	<0.518%	
Altered magnetic- field duration	$T_{D}$	50 ms	26.32 ms	26.32 ms	50 ms	<100 μs	<100 μs
AC amplitude	$B_{0}$	≈39.46 μT					
		Incubator E			Incubator C		
Geomagnetic (DC) field	$B_{x}$	≈8.0 μT			≈−17.5 μT		
	$B_{y}$	≈34.0 μT			≈−8.0 μT		
	$B_{z}$	≈43.0 μT			≈39.0 μT		
	$|\vec{B}|$	≈56.0 μT			≈43.5 μT		

## Data Availability

Not applicable.

## Supplementary Materials

The following supporting information can be downloaded at: https://www.mdpi.com/article/10.3390/bioengineering9100595/s1, Figure S1: Near field of the AC-coil; Figure S2: Far field of the AC-coil; Figure S3: B/I-calibration for both AC-coils; Figure S4: Frequency- and phase response for the calibration; Figure S5: Geo-magnetic background in incubator E; Figure S6: Geo-magnetic background in incubator C; Figure S7: NTC-sensor temperature calibration; Table S1: Devices and components; Table S2: Temperature fluctuations; Table S3: Data summary—single measurements; Table S4: Data Summary—mean values; Raw-Data.xlsx Raw Data; Calibration Certificate: Stefan Meyer Instrument, Fluxmaster; Calibration Certificate: KeithleyDAQ6510; Calibration Certificate 2019-06: Pt100 FPA22L0100 DAkkS.

## Abbreviations

The following abbreviations are used in this manuscript:

MF	Magnetic field
EMF	Electromagnetic field
ELF-MF	Extreme low-frequency magnetic field
PEMF	Pulsed electromagnetic field
TLR	Toll-like receptor
CIT	Cell information therapy
OD	Optical density
IC	Incubator
NTC	Negative temperature coefficient
MSM	Multisensor-module
HEK	Human embryonic kidney
PMMA	Poly(methyl methacrylate), acrylic glass
