# Supplementary material for: A Novel Method to Achieve Precision and Reproducibility in Exposure Parameters for Low-Frequency Pulsed Magnetic Fields in Human Cell Cultures
Source: Bioengineering (Basel). 2022 Oct 21;9(10):595. doi: 10.3390/bioengineering9100595 (PMC9598188; doi:10.3390/bioengineering9100595)
Supplement: Supplementary file 1 [file bioengineering-09-00595-s001.zip › KeithleyDAQ6510.pdf]

KEITHLEY
A Tektronix Company

A Greater Measure of Confidence

TRACEABLE CALIBRATION
DAQ6510, 4455554
Cal Date: 18-FEB-2020
Cal Due: Lab: Kei
KEITHLEY

KEITHLEY INSTRUMENTS, INC * 28775 AURORA RD, CLEVELAND, OHIO - USA * 440-248-0400 * Fax: 440-248-6168 * 1-888-KEITHLEY * www.keithley.com

Calibration Facility: This product was calibrated for Keithley Instruments by
Tektronix (China) Co. Ltd., 1227 Chuan Qiao Road, Pudong New District, Shanghai, China 201206

Calibration Certificate

Certificate No: PC8K1274HF2
Manufacturer: Keithley
Description: Switch Multimeter
Calibration Date: 18-FEB-2020

Revision: 00
Model: DAQ6510
Temperature: 23.0 °C
Date Placed In Service: __________ *

Serial No: 4455554
Humidity: 34 %
Due Date: __________ *

* Optional customer entry fields: The due date may be established by adding the Keithley recommended cal interval stated in the product manual to the "Date placed in service"

Initial Condition: Not applicable, new product **Final Condition:** In Tolerance

- Keithley Instruments, Inc. certifies that the above instrument meets its published measurement specifications.
- This instrument has been calibrated using measurement standards traceable to the International System of Units (SI) through the PRC National Institute of Metrology (NIM), or other National Metrology Institutes (such as NIST, NPL, PTB, etc.).
- The policies and procedures used for the calibration of this product are based upon ANSI/NCSL Z540.1-1994 (R2002).
- The quality system used by the calibration facility is ISO 9001:2015 registered.
- This calibration is a direct comparison of the unit under test to the listed reference standards and did not involve any sampling plans to complete. No allowance has been made for the instability of the test device due to use, time, etc. Such allowances would be made by the customer as needed.
- This calibration certificate shall not be reproduced, except in full, without the written approval of Keithley Instruments, Inc.

Calibration Procedure Used: MANIFEST:Product_Dmm_2012-KeithleyDMMs_Full VERSION:86

Calibration Standards Used:

Manufacturer/Model	Model Description	ID Number	Due Date
Fluke 5725A	Power Amplifier	KI6652	30-Jun-2020
Fluke 5730A	Multifunction Calibrator	KI10627	29-Dec-2020
Fluke 8508A	Digital Multimeter	KI10606	09-Oct-2020
IET Labs 1423-A	Decade Capacitor	KI198481	21-May-2020
IET Labs HACS-Z-A-3E-1uF	Decade Capacitance Substituter	KI198482	21-May-2020
Keithley 3390	Function/Arbitrary Waveform Gen	KI10632	15-Nov-2020

Issued By: [signature]
Quality Director: LI Yongdong

Certified By: Yuzhong Tian
Date Issued: 19-FEB-2020
