# Supplementary material for: A Novel Method to Achieve Precision and Reproducibility in Exposure Parameters for Low-Frequency Pulsed Magnetic Fields in Human Cell Cultures
Source: Bioengineering (Basel). 2022 Oct 21;9(10):595. doi: 10.3390/bioengineering9100595 (PMC9598188; doi:10.3390/bioengineering9100595)
Supplement: Supplementary file 1 [file bioengineering-09-00595-s001.zip › Pt100FPA22L0100.pdf]

Ahlborn Mess und Regelungstechnik GmbH

akkreditiert durch die / *accredited by the*

Deutsche Akkreditierungsstelle GmbH

als Kalibrierlaboratorium im / *as calibration laboratory in the*

Deutscher Kalibrierdienst

DKD

ilac-MRA | DAkkS Deutsche Akkreditierungsstelle D-K-19342-01-00

Kalibrierschein
Calibration Certificate

Kalibrierzeichen
Calibration mark

0105
D-K-19342-01-00
2018-02

Gegenstand
Object

Pt100 Temperaturfühler mit Messgerät
System ALMEMO®

Hersteller
Manufacturer

Ahlborn Mess- und Regelungstechnik GmbH

Typ
Type

Anzeigegerät: **MA2690-8A**
Fühler: **FPA22L0100**

Fabrikat/Serien-Nr.
Serial number

Anzeigegerät: **H18020035**
Fühler: **---**

Auftraggeber
Customer

Sachtleben GmbH
DE-20251 Hamburg

Auftragsnummer
Order No.

4064052

Anzahl der Seiten des Kalibrierscheines **2**
Number of pages of the certificate

Datum der Kalibrierung **20.02.2018**
Date of calibration

Dieser Kalibrierschein dokumentiert die Rückführung auf nationale Normale zur Darstellung der Einheiten in Übereinstimmung mit dem Internationalen Einheitensystem (SI). Die DAkkS ist Unterzeichner der multilateralen Übereinkommen der European co-operation for Accreditation (EA) und der International Laboratory Accreditation Cooperation (ILAC) zur gegenseitigen Anerkennung der Kalibrierscheine.
Für die Einhaltung einer angemessenen Frist zur Wiederholung der Kalibrierung ist der Benutzer verantwortlich.

This calibration certificate documents the traceability to national standards, which realize the units of measurement according to the International System of Units (SI).
The DAkkS is signatory to the multilateral agreement of the European co-operation for Accreditation (EA) and of the International Laboratory Accreditation Cooperation (ILAC) for the mutual recognition of calibration certificates.
The user is obliged to have the object recalibrated at appropriate intervals.

Dieser Kalibrierschein darf nur vollständig und unverändert weiterverbreitet werden. Auszüge oder Änderungen bedürfen der Genehmigung sowohl der Deutschen Akkreditierungsstelle GmbH als auch des ausstellenden Kalibrierlaboratoriums. Kalibrierscheine ohne Unterschrift haben keine Gültigkeit.
This calibration certificate may not be reproduced other than in full except with the permission of both the Deutsche Akkreditierungsstelle GmbH and the issuing laboratory. Calibration certificates without signature are not valid.

Datum<br>*Date*	Leiter / stellv. Leiter des Kalibrierlaboratoriums<br>*Head / Deputy Head of the calibration laboratory*	Bearbeiter<br>*Person in charge*
**20.02.2018**	Dipl. Ing. R. Waldera	N.Wacker N. Wacker

0105
D-K-
19342-01-00
2018-02

Kalibriergegenstand: Pt100- Temperaturfühler mit Messgerät
Auflösung 0,001°C /M00

Kalibrierverfahren: Die Kalibrierung erfolgte durch direkten Vergleich der durch das Bezugsnormal dargestellten Messwerte mit den Messwerten des Prüflings.

Referenznormale: AKF009 Temperaturnormal DAkkS-D-K-15219-01-00-2073

Messbedingungen: Kalibrierung im Flüssigkeitsbad
Eintauchtiefe: 100 mm

Korrekturwerte (ALMEMO®):
NK: 0,119
SK: 0,9972

Umgebungstemperatur:	25 °C	± 2°C
Umgebungsluftfeuchte:	24 %	± 5 %
Luftdruck:	933 hPa	± 10 hPa

Messergebnisse:

Temperatur			
Referenzwert °C	Anzeige Prüfling °C	Abweichung K	Messunsicherheit K
18,065	18,074	0,009	0,040
37,002	37,013	0,011	0,040
44,986	44,989	0,003	0,040
60,004	60,003	-0,001	0,040

Messunsicherheit: Angegeben ist die erweiterte Messunsicherheit, die sich aus der Standardmessunsicherheit durch Multiplikation mit dem Erweiterungsfaktor k = 2 ergibt. Sie wurde gemäß DAkkS-DKD-3 ermittelt. Der Wert der Messgröße liegt mit einer Wahrscheinlichkeit von 95 % im zugeordneten Werteintervall. Ein Anteil für die Langzeitstabilität und das Hystereseverhalten des Kalibriergegenstandes ist nicht enthalten.
