# Supplementary material for: A Novel Method to Achieve Precision and Reproducibility in Exposure Parameters for Low-Frequency Pulsed Magnetic Fields in Human Cell Cultures
Source: Bioengineering (Basel). 2022 Oct 21;9(10):595. doi: 10.3390/bioengineering9100595 (PMC9598188; doi:10.3390/bioengineering9100595)
Supplement: Supplementary file 1 [file bioengineering-09-00595-s001.zip › StefanMeyerInstruments-Fluxmaster.pdf]

Stefan Mayer Instruments
Fluxgate Magnetometers & more

KALIBRIERZERTIFIKAT

Modell: **Fluxgate-Magnetometer FLUXMASTER**
Seriennummer: **508**
Datum der Kalibrierung: 01. Juni 2018
Umgebungstemperatur: 20 °C ± 1 °C
Kalibrierung durchgeführt von: R. Smolenski

Messmittel

Messmittel	Nummer	kalibriert am	kalibriert durch
Helmholtzspule Durchm. 64 cm	26811 PTB 15	1. 10. 2015	PTB Braunschweig
Strommessgerät Keithley 2000	0601390	16. 10. 2017	Keithley Instruments GmbH

Messmethode

Der Sensor des Magnetometers wurde im Zentrum der Helmholtzspule positioniert und entlang der Spulenachse ausgerichtet. Es wurden unterschiedliche Ströme in die Spule eingeprägt. Der Anteil des Erdmagnetfeldes wurde durch Umpolen der Stromquelle und Differenzbildung der Messwerte kompensiert. Der Nullpunktfehler wurde mit Hilfe einer dreifachen Mumetallabschirmung bestimmt.

Messergebnisse

Messbereich	Sollwert +/-0,5%	Messwert vor Kalibrierung	Messwert nach Kalibrierung
1	200,0 µT	200,7 µT +/-0,1 µT	200,0 µT +/-0,1 µT
2	20,00 µT	20,09 µT +/-0,01 µT	20,00 µT +/-0,01 µT
3	2,000 µT	2,007 µT +/-0,005 µT	2,000 µT +/-0,005 µT
Nullpunkt	0 +/-5 nT	2 nT +/-5 nT	0 nT +/-5 nT

Dieses Kalibrierzertifikat dokumentiert die Rückführbarkeit auf nationale Standards.

Dinslaken, den 01. Juni 2018
Stefan Mayer Instruments

P. Glasenapp

(Dr. Philipp Glasenapp)

Stefan Mayer Instruments
GmbH & Co. KG. Wallstr. 7
46535 Dinslaken. Germany
Tel./Fax +49 2064 479762/3
mail@stefan-mayer.com

Stefan Mayer Instruments GmbH & Co. KG
Wallstraße 7 • D-46535 Dinslaken • Deutschland
Telefon: +49 (0)2064 479762
Telefax: +49 (0)2064 479763
www.stefan-mayer.com • mail@stefan-mayer.com

AG Duisburg HRA 12336 • Persönlich haftende Gesellschafterin:
Stefan Mayer Verwaltungs GmbH • Sitz Dinslaken • AG Duisburg HRB 30336
Geschäftsführer: Dipl.-Phys. Dr. Stefan Mayer
USt-IdNr.: DE 315972918 • WEEE-Reg-Nr.: DE 67865463
Commerzbank AG • IBAN: DE72 3564 0064 0610 5589 00 • BIC: COBADEFFXXX
