# Supplementary material for: A Novel Method to Achieve Precision and Reproducibility in Exposure Parameters for Low-Frequency Pulsed Magnetic Fields in Human Cell Cultures
Source: Bioengineering (Basel). 2022 Oct 21;9(10):595. doi: 10.3390/bioengineering9100595 (PMC9598188; doi:10.3390/bioengineering9100595)
Supplement: Supplementary file 1 [file bioengineering-09-00595-s001.zip › Supplement.pdf]

S1 Technical System

S1.1 Devices and components

The devices and components used in the system are listed in Table S1.

Table S1: Devices and components. Summary of the devices and components, which are used in the exposure system.

Device/ Component	ID and Manufacturer	Comment
Function Generator	33511B, Keysight Technologies Inc. 1400 Fountaingrove Parkway, Mailstop 1 USM, Santa Rosa, CA 5403, USA	arbitrary function generator
Amplifier	DCP 130/60 HSR, Servowatt Leistungselektronik GmbH, 70839 Gerlingen, Germany	4-quadrant amplifier, 200 kHz, current regulated
Coil	Rechteckspule_03/04, Sachtleben GmbH, Martinistraße 64, 20251 Hamburg, Germany	custom-made
Oscilloscope	PicoScope 4444, Pico Technology, James House, Colmworth Business Park, St. Neots Cambridgeshire, PE19 8YP United Kingdom	differential oscilloscope
Data Logger	ALMEMO 809, Ahlborn Mess- und Regelungstechnik GmbH, Eichenfeldstraße 1, 83607 Holzkirchen, Germany	9 slots, DAkkS calibrated
Multisensor-modul	FHAD 46-C2, Ahlborn Mess- und Regelungstechnik GmbH, Eichenfeldstraße 1, 83607 Holzkirchen, Germany	measures temperature and humidity within the incubator, factory calibrated
NTC-sensor	TS-NTC-103A, B & B Sensors, Heinrich-Hertz-Straße 4, 78166 Donaueschingen, Germany	measures temperature within the incubator, calibrated with calibration bath
CO_2-sensor	SPRINTIR-WF-20, Gas Sensing Solutions Ltd., 60-62 Grayshill Road, Westfield Industrial Estate, Cumbernauld, United Kingdom, G68 9HQ	calibrated via CO_2-Controller
CO_2-controller	CO_2-Controller 2000, PeCon GmbH, Ziegeleistrasse 50, 89155 Erbach, Germany	CO_2 supply for 2 incubators, auto-calibration function
Thermostat	Julabo GmbH, Gerhard-Juchheim-Strasse 1, 77960 Seelbach, Germany	Temperature regulator for one incubator

S2 Magnetic Field Generation

S2.1 Magnetic field distribution of the rectangular coil

The construction of rectangular coils is based on a calculation of the magnetic field distribution that meet the requirements. This was done by an analytical solution of the Biot-Savart law for a rectangular coil geometry in air ($\mu_r = 1$).

Figure S1 shows the geometry from 2 side views and the top views. The top view was calculated for 2 different x-y-planes at $z_1 = -20.0\,mm$, $z_2 = 0.0\,mm$. These planes are the bottom of the 2 stacked 24-well plates[1]. Figure S2 shows the magnetic field distribution in the far field range of the coil up to $2\,m$ away. Here, the field intensity drops by a factor of 10^6 for a distance $\geq 2\,m$ and by a factor of 10^5 for $\geq 1\,m$ relative to the center. For verification, the magnetic field was measured along the x-, y- and z-axis at definite distances. The result is shown in Figure S2D and demonstrates that the measurement fits the calculation.

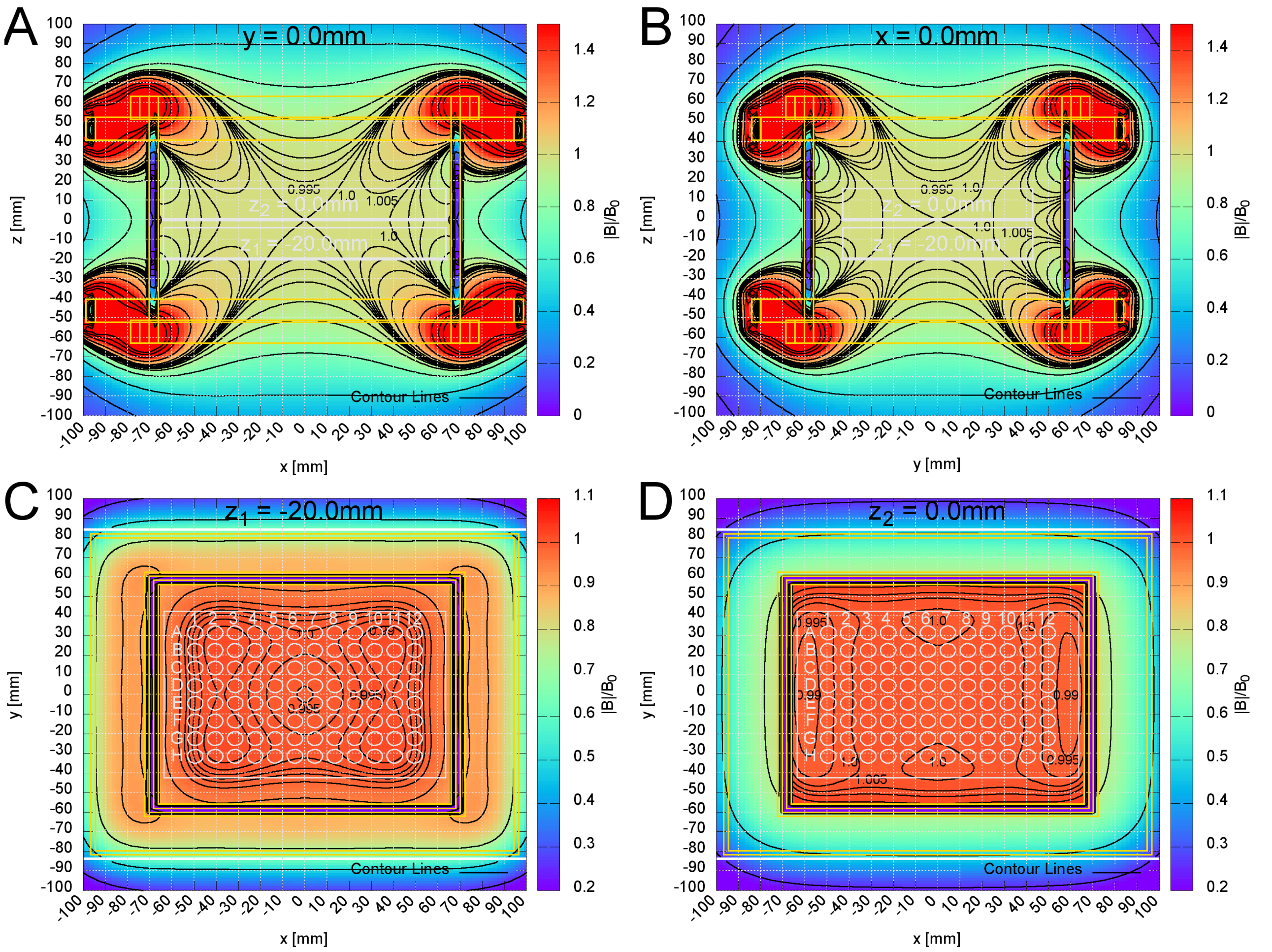

Figure S1: Near field of the AC-coil. The figure shows the magnetic field distribution of the rectangular coil in the near field as a heatmap from different views. The figures at the bottoms shows the field distribution over the 96-well-plates for the inner region at different z-layers. Top figures show side views as well as measurements for verification. Contour lines are shown in black $(0.99, 0.995, 1.0, 1.005)$. $z_1 = -20.0\,mm$ and $z_2 = 0.0\,mm$ indicate position of the bottom of the 96/24-well plates.

S2.2 $\frac{B}{I}$-calibration slope factor

A spacial reference point is required to calibrate the magnetic field and the input current. This point can be arbitrary chosen. A good and easily accessible choice would be the center of the coil in z-direction. At this point the x- and y-components of the field vanishes. Using a calibrated fluxgate magnetometer (Fluxmaster, Stefan Mayer Instruments GmbH & Co. KG, Hans-Böckler-Straße 21c, 46535 Dinslaken, Germany) and a precise multimeter (DAQ6510 Multimeter, Tektronix Inc., 14150 SW Karl Braun Drive, P.O. Box 500 Beaverton, OR 97077,

[1]The standard 24-well plate is higher than the 96-well plate.

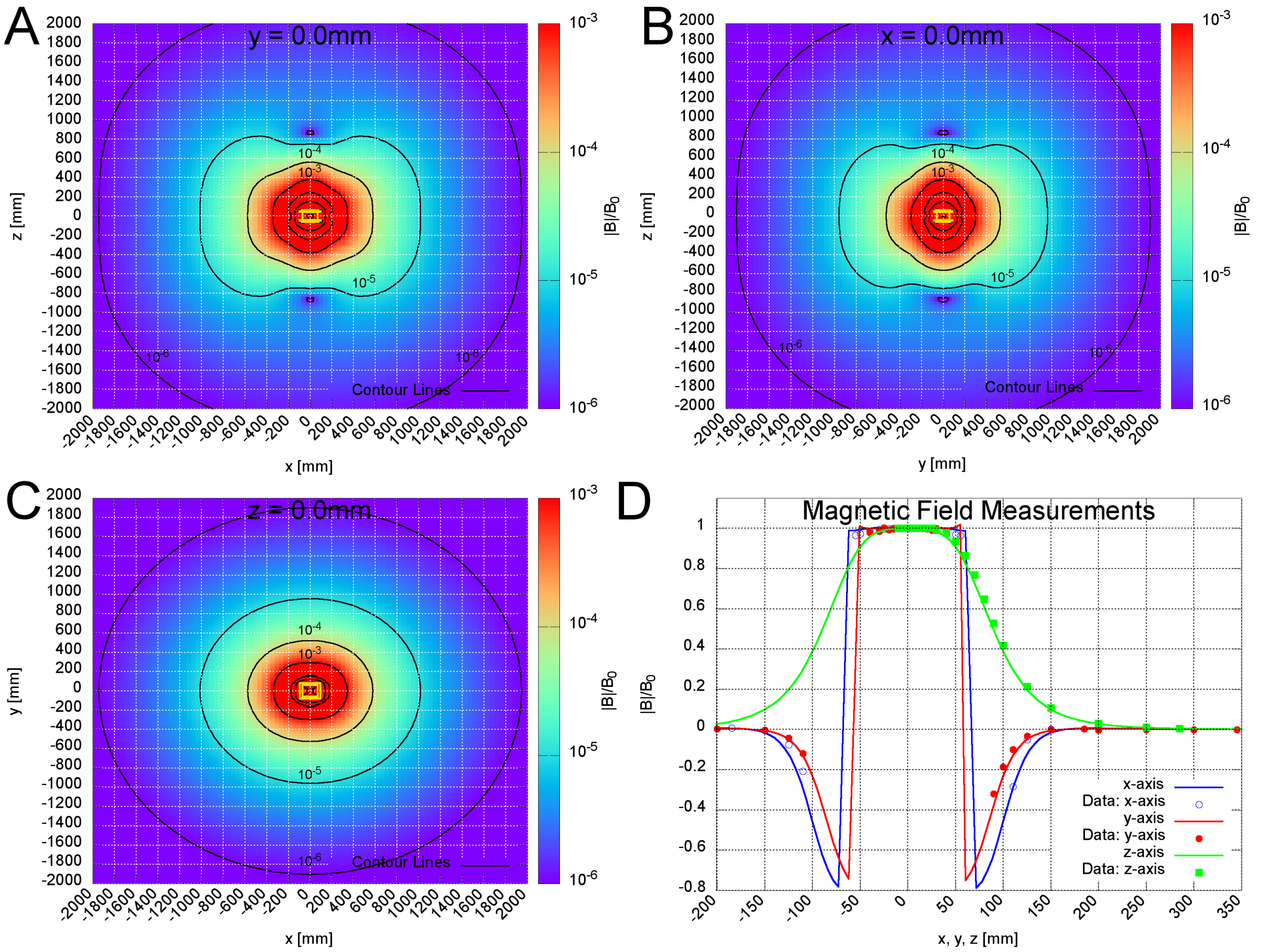

Figure S2: Far field of the AC-coil. Similar to Figure S1 the figure shows the far field range of the magnetic field intensity up to 2m from the center of the coil. Subfigure D verifies the calculation with measured magnetic fields values (in z-direction) along the x-, y- and z-axis at several distances.

USA) for the current measurement the calibration can be easily done for the coil. A power supply (ABC 25-4DM, Kepco Inc., 131-38 Sanford Avenue, Flushing, NY 11355, USA) with low noise and high stability provides the electric current. Measuring the magnetic field versus the electric current for several electric currents allows a linear fit of the data:

$$B(I) = B_0 + K \cdot I \tag{S1}$$

This leads to the $\frac{B}{I}$-ratio K. The offset constant absorbs the ambient geomagnetic field and has no meaning for the calibration. For both coils, the exact $\frac{B}{I}$-ratios and the corresponding coefficients of determination R^2 are shown in Figure S3 A and B.

S2.3 Coil-Amplifier calibration

Furthermore, the amplifier and coil needs to be frequency corrected by accounting for the frequency and phase response of the system. A calibration beyond the specified frequency range and close to the power limit of the amplifier (DCP 130/60 HSR, Servowatt GmbH, Gerlingen, Germany) requires a 2-dimensional calibration for the frequency and the input voltage amplitude. Figure S4 shows the frequency response of the coil-amplifier system for several input voltage amplitudes. Here, the input voltage and the coil current were measured simultaneously with a differential oscilloscope. The current was measured via a shunt resistance integrated in the amplifier. Both signals were analyzed by a self-made python software, which extracts

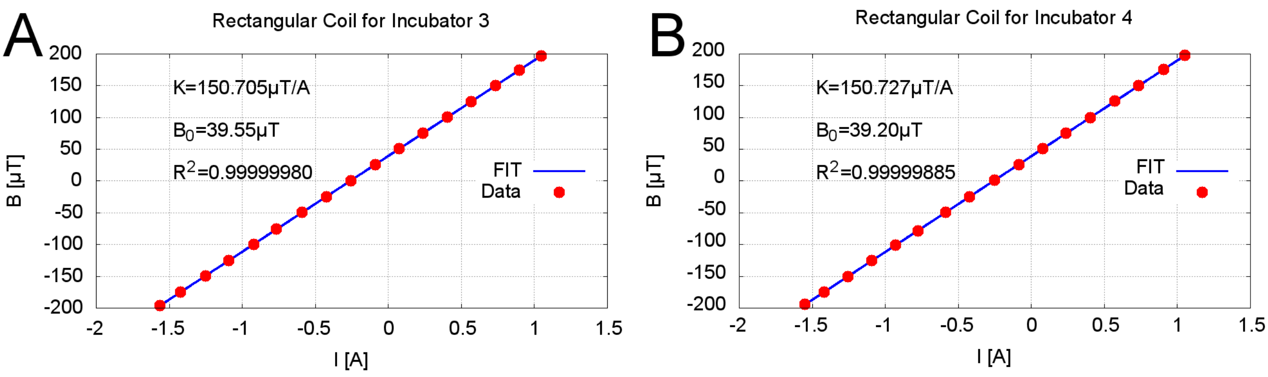

Figure S3: $\frac{B}{I}$-calibration, for both AC-coils including the linear regression (FIT) according Equation S1. In addition to the slope K the offset B_0 and the coefficient of determination R^2 are also shown.

the amplitude ratio and the phase shift between both voltages as well as the quality-index of the measurement with a R^2-value calculation of the fit and automates the measurement. Figure S4 displays amplitude- and phase-measurements color-coded depending on the R^2-values. Subfigures on the left show the magnetic amplitude and subfigures on the side show the phase response. At roughly $100\,kHz$ both the intensity of the magnetic field and the phase shift start to decrease. This sounds reasonable for an amplifier effect, which is operating beyond its specified frequency range of $200\,kHz$. The sinusoidal signal was generated by a function generator (33511B, Keysight Technologies, Santa Rosa, CA 5403, USA) and is connected to the amplifier (DCP 130/60 HSR) via a $50\,\Omega$-termination impedance.

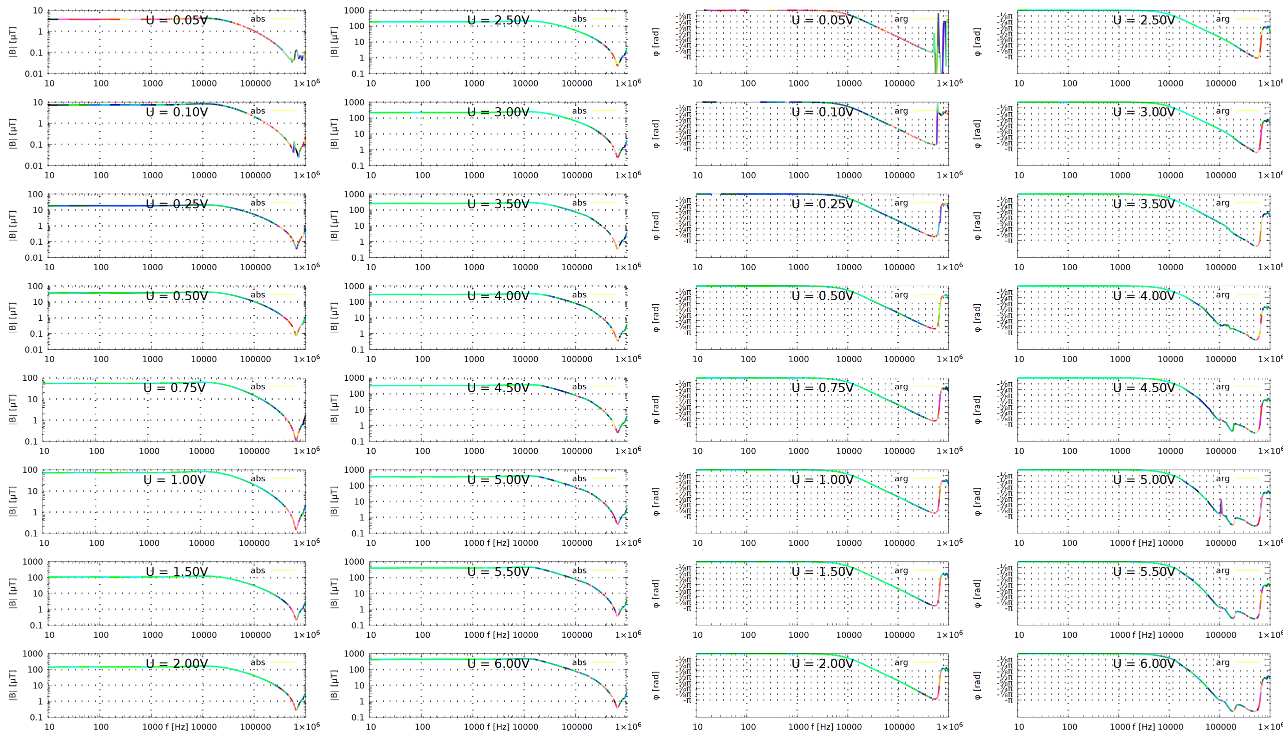

Figure S4: Frequency- and phase responses for the calibration of the rectangular coil for several amplitudes up to $1\,MHz$. Amplitude responses are shown on the left and phase responses on the right.

S2.4 Input-Signal Transformation

Generating any magnetic field signal in the ELF-MF/PEMF range requires a predistortion of the input signal to compensate for the intrinsic amplitude and phase shifts. This requires the calibration of the complete amplifier-coil system. This is done by transforming the input signal into frequency space via an FFT (Fast Fourier Transform) followed by multiplication with the calibrated frequency and phase response function of the amplifier and coil. Finally, the spectrum is transformed back into the time frame via the inverse FFT and the signal is uploaded to the function generator.

S3 Magnetic Field Measurements

S3.1 Geomagnetic Background Field

In order to measure the constant background field within the incubation space at the positions of the 96-well-plate, we have constructed an automatic magnetic field scanner travel drive that enables measurements in the x-y-plane with a calibrated magnetic field sensor (RM3100, PNI, 2331 Circadian Way, Santa Rosa, CA 95407, USA). The active sensor area has dimensions of roughly $6\,mm \times 6\,mm \times 6\,mm$ and was calibrated versus a DAkkS calibrated fluxgate magnetometer from Stefan Meyer instruments (Fluxmaster) in all 3 directions with a self-made 3D-coil (not shown here). Similar to the calibration of the AC-coil in section S2.2 a Kepco ABC power supply was used to provide a stable electric current. In Figure S5 and Figure S6 all magnetic field components including absolute value are shown for both incubators E and C, respectively. The magnetic field in incubator E (exposure) is more inhomogeneous compared to that in incubator C (control).

The grid was set to $10\,mm$ and the data was interpolated via a 3rd-order 2D-polynomial fit of the form

$$P(x,y) \equiv A_1x^3 + A_2x^2y + A_3xy^2 + A_4y^3 + B_1x^2 + B_2xy + B_3y^2 + C_1x + C_2y + D, \qquad \text{(S2)}$$

whereas $\{A_i, B_i, C_i, D\}$ are the fit constants. The fit was applied to each magnetic field component and also to the absolute value with the Levenberg-Marquardt method. Coefficients of determination are shown at the top of each subfigure in Figure S5 and Figure S6. The coil, including the 96-well plate, was pushed to the right lower corner from the top view in order to guarantee a reproducible positioning of the sample in the local geomagnetic field.

S3.2 Magnetic Field Exposure Conditions

The total magnetic field at each position of the 96-well plate is the sum of the constant geomagnetic background and the alternating field. It is defined as

$$\vec{B}(\vec{r},t) = \vec{B}_{geo}(\vec{r}) + \vec{B}_{AC}(\vec{r},t). \qquad \text{(S3)}$$

Here, $\vec{r}$ is the position vector and $\vec{B}_{AC}(\vec{r},t) = \vec{B}_0(\vec{r})\,g(t)\hat{e}_z$ is the alternating field in z-direction (vertical) with amplitude distribution $\vec{B}_0(\vec{r})$ and signal shape $g(t)$. The latter is normalized by $\max_{0\le t\le T}|g(t)| = 1$ with $T \equiv \frac{1}{f}$ and $\vec{B}_0(\vec{r})$ can be approximated by $\vec{B}_0(\vec{r})\big|_{|\vec{r}|=0} \approx B_0\hat{e}_z$ close to the center.

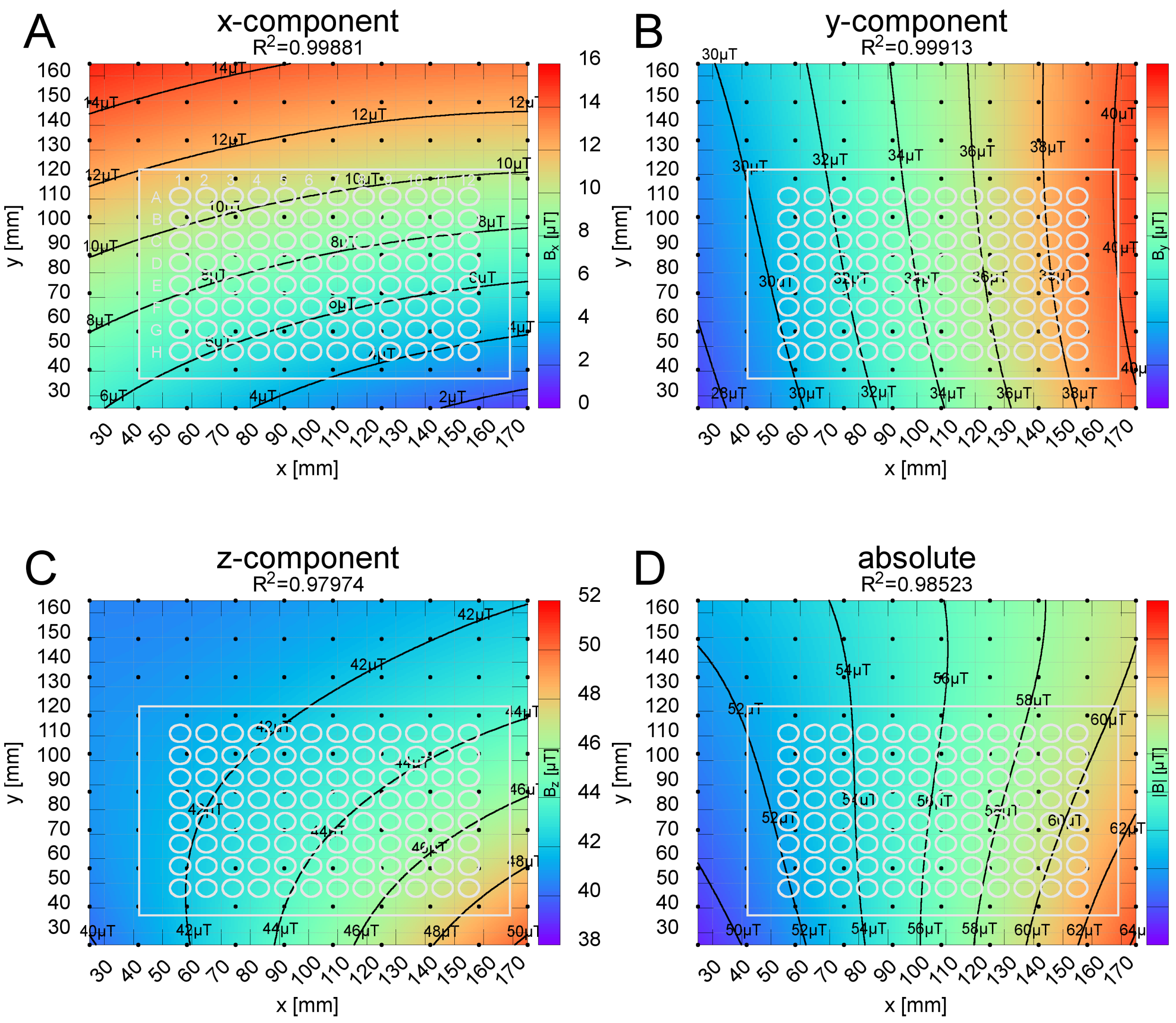

Figure S5: Geomagnetic background in incubator E (exposure). Subfigure A-C show the distribution of the x-, y- and z-components of the magnetic field in the x-y plane at $z = 43\,mm$ over the incubator bottom where the cell culture was placed within the coil. Data was taken via an automatized self-made travel drive scanning the x-y-layer with a calibrated magnetic field sensor (RM3100 from PNI). Subfigure D summarizes the total magnetic field. The heatmap was created by 2D-polynomial fit (Equation S2) to the data points (positions are labeled by black dots and contours by black lines with corresponding values). The fit was done by the Levenberg-Marquardt method using GnuPlot. Coefficients of determination R^2 for the fit are shown at the top of each picture.

S3.3 Electric Field Induction

We have estimated the induced electric field strength E_Φ in the cell culture medium in order to exclude electric side effects:

$$E_\Phi(\vec{r}, t) = -\frac{|\vec{r}|}{2} \frac{\partial B_{AC}(\vec{r}, t)}{\partial t} \qquad \text{(S4)}$$

The well diameter of a 96-well plate is $\varnothing 6.8mm$.

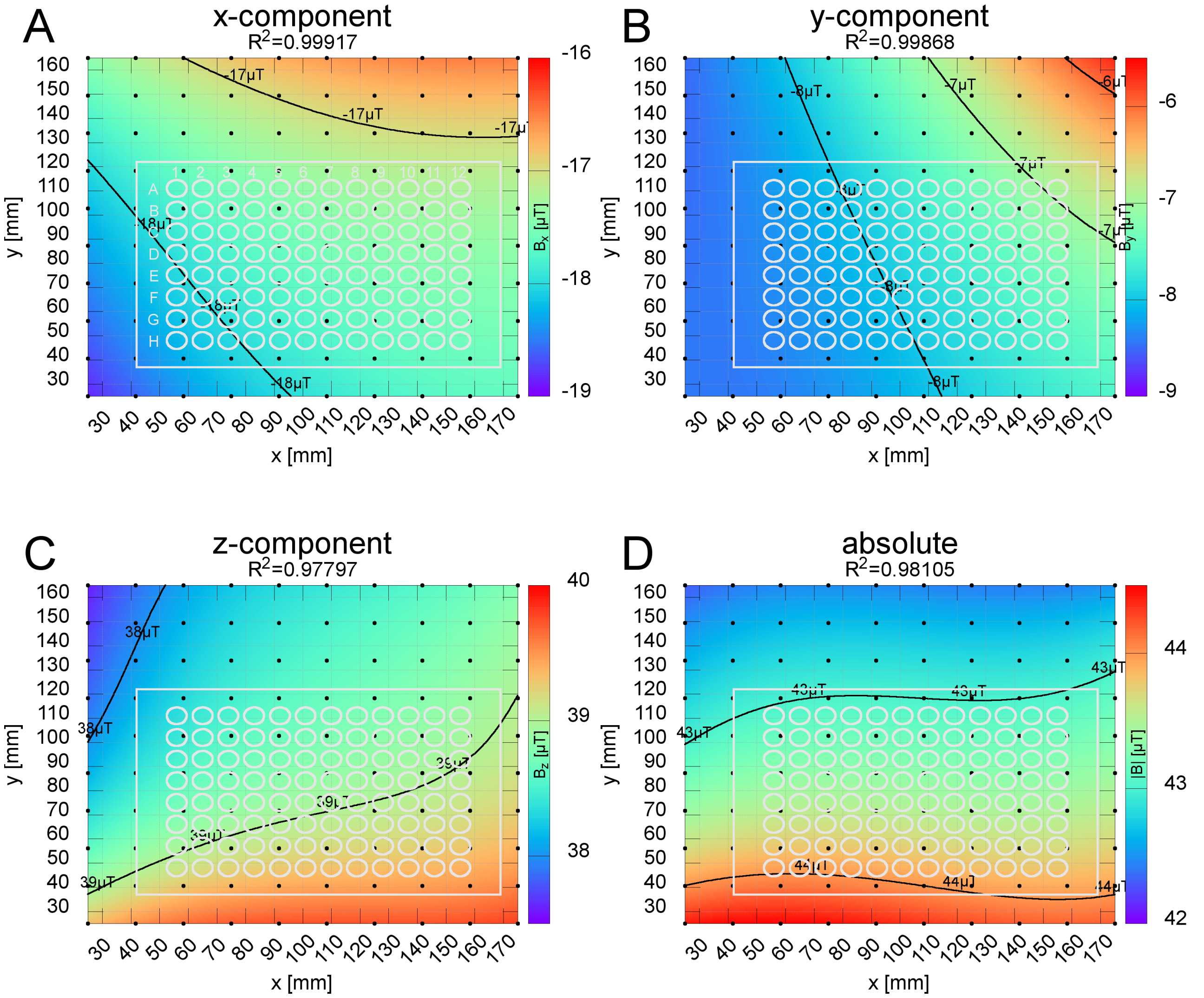

Figure S6: Geomagnetic background in incubator C (control). See also caption of Figure S5. Here, the field is much more homogeneous as in incubator E (exposure) in Figure S5. Coefficients of determination for the fit are shown at the top of each picture.

S4 Temperature, humidity and CO_2-concentration

S4.1 Temperature Calibration of the NTC-Sensors

The calibration of temperature sensors is necessary for a precise adjustment of temperature inside the incubators, especially in the vicinity to 37°C. Figure S7 shows the temperature curves of 2 NTC sensors and the calibrated reference Pt100 sensor (blue). All sensors show a good agreement at 25°C but not at 37°C. The temperature for the calibrated Pt100 and the NTC sensors from Figure S7A were averaged over each plateau and plotted in Figure S7B and C. The linear fit has the form

$$T_{real}(T_{nominal}) = \alpha(T_{nominal} - T_0) \tag{S5}$$

Both fit parameters α and T_0 are displayed in the Figure S7B and C, respectively and were programmed into the data logger (ALMEMO-plug).

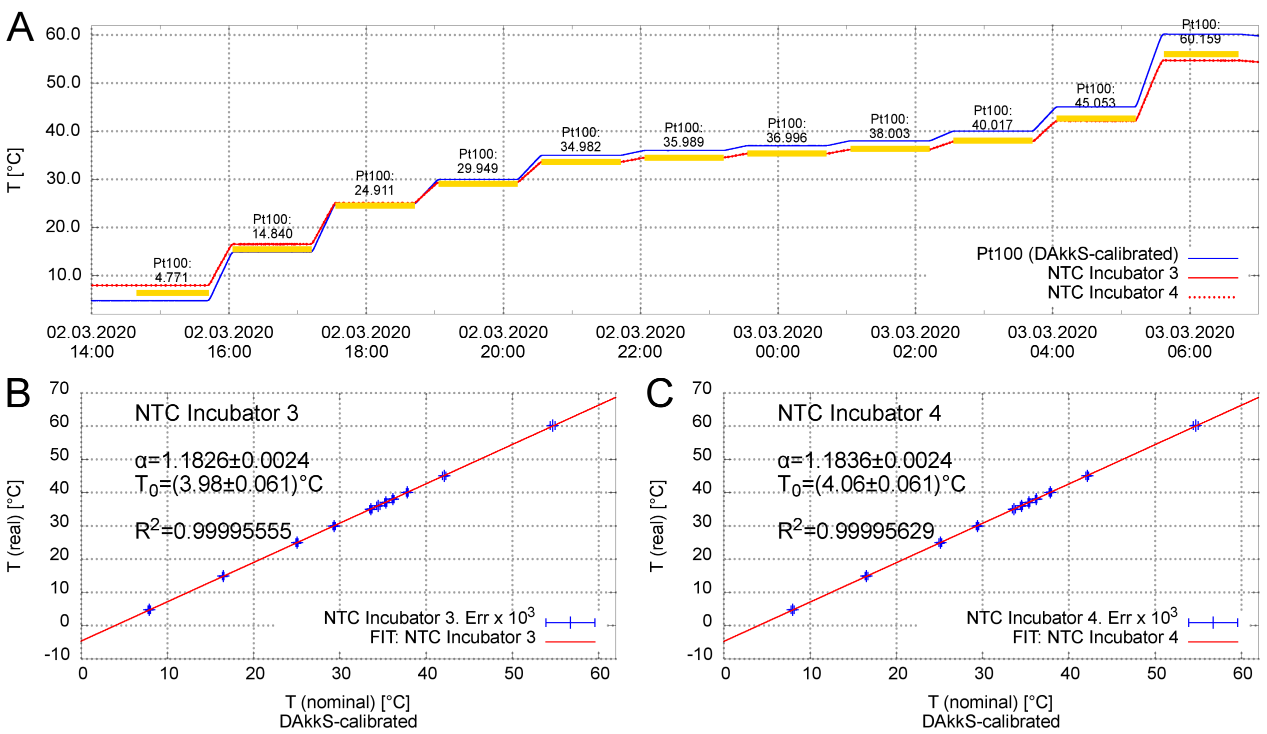

Figure S7: NTC-sensor temperature calibration. Temperature curves within an calibration thermostat are shown in subfigure A for all NTC-sensors. The calibration coefficients in subfigures B and C result from the linear regression of the nominal (calibrated Pt100) and real (NTC) value for the NTC-sensors. Subfigure B corresponds to incubator E and subfigure C to incubator C, respectively.

S4.2 Fluctuations in temperature, humidity and CO_2-concentration

Fluctuations in temperature, humidity and CO_2-concentration can be estimated by the mean, standard deviation, minimum and maximum values over a defined duration, here exemplary for experiments without distortions in the time period between 08.12.2022, 2pm and 10.12.2022, 10am. The total experiment duration includes the incubator opening and thus gives information of the total temperature, humidity and CO_2 loss within the incubators. Characteristic values are summarized in Table S2.

Table S2: Temperature fluctuations. Summary of the mean, standard deviation, minimum and maximum values for temperature, relative humidity and CO_2 in incubator E (IC E) and C (IC C) for the complete experiment and the time after opening and equilibration until the end of the experiment (08.12.2022 2pm - 10.12.2022 10am), which is labeled in Figure 4 as well.

		**total**		**08.12. 2pm-10.12. 10am**	
**Quantity**	**Inkubator**	**IC E**	**IC C**	**IC E**	**IC C**
T [°c]	MEAN	36.947	36.967	37.008	37.010
	STD	0.282	0.241	0.032	0.038
	MIN	26.53	29.45	36.97	36.93
	MAX	37.16	37.21	37.16	37.16
	MAX-MIN	10.63	7.76	0.19	0.23
rH [%]	MEAN	94.17	90.69	92.55	89.82
	STD	2.97	1.46	0.12	0.58
	MIN	27.6	58.3	92.2	88.5
	MAX	98.9	94.1	92.9	91.0
	MAX-MIN	71.30	35.80	0.70	2.50
[CO_2] [%]	MEAN	4.61	4.89	4.57	4.91
	STD	0.20	0.12	0.02	0.02
	MIN	1.23	1.30	4.50	4.85
	MAX	8.12	5.55	4.65	4.97
	MAX-MIN	6.89	4.25	0.15	0.12

S5 Data calculation and tables

The raw data needs to be normalized to be comparable. The procedure is shown in the subsequent subsection S5.1.

S5.1 Data calculation

The normalisation of the inflammatory response measurements for the OD620-QUANTI-Blue^{TM} and the OD750 for the total protein concentration by 4 independent OD620/OD750-ratios are defined by

$$Q_{E/C,LPS}^{(i,j)} := \frac{OD_{E/C,LPS}^{(620,i,j)}}{OD_{E/C,LPS}^{(750,i,j)}}, \tag{S6a}$$

$$Q_{E/C}^{(i,j)} := \frac{OD_{E/C}^{(620,i,j)}}{OD_{E/C}^{(750,i,j)}}, \tag{S6b}$$

for the control (C) and exposed (E) measurement with and without LPS and for the j-th replicate ($j \in \{1, \ldots, n\}$, $n = 5$) within the i-th repetition ($i \in \{1, \ldots, N\}$). Subtracting the background SEAP values $Q_E^{(i,j)}$ and $Q_C^{(i,j)}$, respectively, then the following 2 differences can be defined

$$\Delta Q_C^{(i,j)} := Q_{C,LPS}^{(i,j)} - Q_C^{(i,j)}, \tag{S7a}$$

$$\Delta Q_E^{(i,j)} := Q_{E,LPS}^{(i,j)} - Q_E^{(i,j)}. \tag{S7b}$$

Averaging of all replicates (here $j = 1, ..., n$, $n = 5$) for both differences 7a and 7b, the ratios of the inflammatory response can be defined by:

$$R_{IR}^{(i)} := \frac{\overline{\Delta Q_E^{(i)}}}{\overline{\Delta Q_C^{(i)}}} \tag{S8a}$$

$$\Delta R_{IR}^{(i)} := R_{IR}^{(i)} \sqrt{\left(\frac{\sigma\left(\Delta Q_E^{(i)}\right)}{\overline{\Delta Q_E^{(i)}}}\right)^2 + \left(\frac{\sigma\left(\Delta Q_C^{(i)}\right)}{\overline{\Delta Q_C^{(i)}}}\right)^2}, \tag{S8b}$$

Here, the 'bar' denotes the mean-value and $\sigma(\cdot)$ the standard deviation (SD) and $R_{IR}^{(i)}$ is the inflammatory response and $\Delta R_{IR}^{(i)}$ the error of R by propagation of uncertainties. $R_{IR}^{(i)}$ and $\Delta R_{IR}^{(i)}$ are listed in Table S3 for all tested conditions and their repetitions.

Finally, for the overall inflammatory effect, R_{IR} has been averaged over the number of repetitions N. This leads to the mean $\overline{R}_{IR}$ including the SD $\sigma(R_{IR})$ and $\Delta \overline{R}_{IR}$ and summarized in Table S4. The latter was calculated by propagation of errors for the mean:

$$\Delta \overline{R}_{IR} = \frac{1}{N} \sqrt{\sum_{i=1}^{N} \left(\Delta R_{IR}^{(i)}\right)^2} \tag{S9}$$

S5.2 Data tables

The measured data of the ratio $R_{IR}^{(i)}$ for all conditions and repetitions is summarized in Table S3.

Table S3: Data Summary - single measurements. Measured $R_{IR}^{(i)}$ data for all conditions and repetitions including the Sham. Here, $i \in \{1, \dots, N\}$ represents i-th repetition and n the number of replicates for each repetition.

Cond.	i	n	$\overline{\Delta Q_C^{(i)}}$	$\sigma(\Delta Q_C^{(i)})$	$\frac{\sigma(\Delta Q_C^{(i)})}{\overline{\Delta Q_C^{(i)}}}$	$\overline{\Delta Q_E^{(i)}}$	$\sigma(\Delta Q_E^{(i)})$	$\frac{\sigma(\Delta Q_E^{(i)})}{\overline{\Delta Q_E^{(i)}}}$	$R_{IR}^{(i)}$	$\Delta R_{IR}^{(i)}$
Sham	1	5	0.323	0.034	0.105	0.322	0.018	0.057	0.998	0.119
	2	5	0.309	0.031	0.101	0.358	0.041	0.116	1.156	0.177
	3	5	0.285	0.018	0.065	0.290	0.025	0.085	1.017	0.108
	4	5	0.402	0.081	0.203	0.426	0.110	0.258	1.060	0.348
	5	5	0.360	0.027	0.075	0.364	0.066	0.181	1.010	0.197
A: 10 Hz, 50%	1	5	0.315	0.070	0.223	0.189	0.033	0.175	0.598	0.169
	2	5	0.328	0.029	0.089	0.221	0.051	0.233	0.674	0.168
	3	5	0.418	0.042	0.100	0.254	0.019	0.075	0.608	0.076
	4	5	0.390	0.030	0.076	0.248	0.013	0.052	0.635	0.059
	5	5	0.400	0.089	0.221	0.268	0.018	0.065	0.670	0.155
	6	5	0.351	0.049	0.139	0.220	0.062	0.281	0.627	0.197
	7	5	0.420	0.041	0.099	0.263	0.019	0.071	0.626	0.076
	8	5	0.747	0.067	0.090	0.503	0.035	0.070	0.674	0.077
B: 19 Hz, 50%	1	5	0.198	0.036	0.182	0.194	0.044	0.225	0.981	0.284
	2	5	0.318	0.012	0.036	0.302	0.021	0.070	0.952	0.075
	3	5	0.270	0.007	0.027	0.261	0.011	0.043	0.968	0.049
	4	5	0.494	0.023	0.047	0.469	0.043	0.093	0.949	0.099
C: 10 Hz, 26%	1	5	0.311	0.019	0.062	0.282	0.028	0.101	0.909	0.108
	2	5	0.335	0.016	0.047	0.317	0.014	0.046	0.946	0.062
	3	5	0.393	0.036	0.092	0.362	0.018	0.050	0.920	0.096
D: 19 Hz, 95%	1	5	0.333	0.045	0.137	0.208	0.022	0.106	0.625	0.108
	2	5	0.356	0.026	0.073	0.265	0.023	0.086	0.744	0.084
	3	5	0.412	0.052	0.127	0.316	0.015	0.046	0.766	0.103
	4	5	0.491	0.031	0.064	0.383	0.027	0.072	0.780	0.075
E: CIT#81	1	5	0.316	0.023	0.071	0.235	0.011	0.047	0.742	0.063
	2	5	0.430	0.024	0.056	0.315	0.038	0.120	0.734	0.097
	3	5	0.427	0.062	0.146	0.327	0.011	0.035	0.764	0.115
	4	5	0.459	0.032	0.071	0.331	0.027	0.081	0.722	0.078
F: CIT#96	1	5	0.263	0.039	0.149	0.243	0.010	0.040	0.921	0.142
	2	5	0.410	0.053	0.130	0.370	0.033	0.089	0.904	0.142
	3	5	0.260	0.019	0.073	0.249	0.018	0.072	0.954	0.098

Table S4: Data Summary - Mean values. Summary of the mean value for the inflammatory response $\overline{R}_{IR}$ readout from Table S3 (mean) including the standard deviation and the p-value of the Shapiro-Wilk and One-Way ANOVA-analysis including the Dunnett test q-value. One-way-ANOVA p-value color indicated the significance corresponding the asterisk: $p < 0.0001^{****}$, $p < 0.001^{***}$; $p < 0.01^{**}$; $p < 0.05^{*}$. Additionally, a Cohan's d-test was done with the effects size rating by the Bravais-Pearson-correlation coefficient $r \leq 0.1$ weak, $r \leq 0.3$ medium and $r \geq 0.5$ strong effect.

		Infl. Response			Shapiro-Wilk		1-Way-ANOVA			Cohan		
Condition	N	$\overline{R}_{IR}$	$\sigma(R_{IR})$	$\Delta\overline{R}_{IR}$	p-value	passed $p \geq 0.05$	p-value	sign.	Dunnett q-value	d	r	size
Sham	5	1.048	0.058	0.093	0.112	yes	-	-	-	-	-	-
A: 10 Hz, 50 %	8	0.639	0.028	0.047	0.194	yes	< 0.0001	****	17.19	9.852	0.979	strong
B: 19 Hz, 50 %	4	0.962	0.013	0.078	0.513	yes	0.0254	*	3.07	1.923	0.691	strong
C: 10 Hz, 26 %	3	0.925	0.015	0.052	0.588	yes	0.0025	**	4.04	2.553	0.777	strong
D: 19 Hz, 95 %	4	0.729	0.061	0.047	0.118	yes	< 0.0001	****	11.40	4.157	0.900	strong
E: CIT#81	4	0.741	0.015	0.045	0.840	yes	< 0.0001	****	10.98	4.022	0.894	strong
F: CIT#96	3	0.926	0.021	0.074	0.679	yes	0.0028	**	3.99	1.392	0.559	strong
